# MicroRNAs and immunotherapy in testicular germ cell tumors: opportunities and challenges for modulation of the immune microenvironment

**DOI:** 10.3389/fimmu.2026.1847225

**Published:** 2026-06-11

**Authors:** Alejandro Lopez-Saavedra, José Díaz-Chávez, Miguel Angel Jimenez-Ríos, Anna Scavuzzo

**Affiliations:** 1 Tecnologico de Monterrey, Escuela de Medicina y Ciencias de la Salud, Ciudad de México, Mexico; 2 Advanced Microscopy Applications Unit (ADMiRA)-Instituto Nacional de Cancerología, Mexico City, Mexico; 3 Instituto Nacional de Cancerología, Ciudad de México, Mexico; 4 Department of Urology, Instituto Nacional de Cancerología, Mexico City, Mexico; 5 Universidad Autonoma de Mexico, Faculty of Medicine, Estudios de Posgrado, Universidad Autonoma de Mexico, Universidad Nacional Autonoma de Mexico (UNAM), Mexico City, Mexico

**Keywords:** immunotherapy, microRNAs, testicular germ cell tumors, tumor immune microenvironment, tumor mutational burden

## Abstract

Testicular germ cell tumors (TGCTs) are the most common solid malignancies in young men and, despite high cure rates with cisplatin-based multimodal therapy, a clinically relevant subset of patients develops relapsed, platinum-refractory, or treatment-resistant disease with limited salvage options and substantial long-term morbidity. This unmet need has renewed interest in immunotherapy, yet the experience in TGCT has been notably less successful than in other malignancies. Although TGCTs often display PD-L1 expression, tumor-infiltrating lymphocytes, and other features suggestive of immune engagement, immune checkpoint inhibitors have produced largely disappointing results in unselected patients, underscoring the complexity of immune regulation in this immune-privileged disease. Recent conceptual advances place microRNAs (miRNAs) at the center of this problem. Beyond their established diagnostic and prognostic value, miRNAs are increasingly recognized as upstream regulators of immune checkpoints, antigen-presentation pathways, cytokine signaling, macrophage polarization, dendritic-cell and T-cell function, and extracellular-vesicle-mediated tumor–immune crosstalk. This positions miRNAs not only as biomarkers of disease activity, but also as plausible determinants of immunotherapy sensitivity or resistance. At the same time, the field remains marked by important controversies: PD-L1 expression alone is an unreliable predictor of response; the relative contribution of low tumor mutational burden, testicular immune privilege, and microenvironmental suppression remains unresolved; and many proposed TGCT-associated miRNAs with an immunoregulatory role are still inferential rather than causally validated in disease-specific models. Accordingly, major gaps persist in defining which miRNAs are true functional drivers of immune escape in seminomatous versus non-seminomatous TGCT, how they interact with therapy-induced tumor evolution, and which delivery platforms can achieve safe, tumor-directed modulation. We argue that the next phase of the field should move beyond descriptive biomarker studies toward mechanism-based, TGCT-specific translational strategies integrating miRNA mimics, antagomirs, extracellular vesicles, or miRNA-augmented cellular therapies with immune-priming approaches such as epigenetic therapy, radiotherapy, vaccines, or CAR-T platforms. Such a framework could enable more precise biological stratification and improve the efficacy, durability, and tolerability of immunotherapy in refractory TGCT.

## 1 Introduction

Testicular germ cell tumors (TGCTs) are the most common solid malignancies in young men, arising during a life stage in which preservation of fertility, endocrine function, long-term health, and quality of life is particularly critical (1). Although contemporary multimodal management—centered on orchiectomy, cisplatin-based chemotherapy, and selected use of radiotherapy or retroperitoneal lymph node dissection—has achieved remarkable cure rates, a clinically important subset of patients still develops relapsed, refractory, or platinum-resistant disease with poor outcomes and limited therapeutic alternatives (2–4). This problem is especially relevant because survivors of TGCT also face substantial long-term treatment-related morbidity, underscoring the importance of identifying more precise and less toxic strategies.

In parallel, immunotherapy has transformed the treatment landscape of several cancers by restoring anti-tumor immune surveillance and generating durable responses in selected settings (5–7). However, in TGCT, this promise has not yet been fulfilled. Despite frequent expression of immune checkpoint molecules such as PD-L1, the presence of tumor-infiltrating lymphocytes, and a compelling biological rationale for immune-based interventions, clinical trials with immune checkpoint inhibitors in unselected refractory TGCT have produced largely disappointing results (8–11). This apparent paradox suggests that TGCT immune responsiveness is governed by a more complex regulatory network than checkpoint expression alone, involving immune privilege, low tumor mutational burden, context-dependent antigenicity, and a highly specialized tumor microenvironment.

Within this framework, microRNAs (miRNAs) emerge as particularly attractive molecules at the interface of tumor biology and immune regulation. Beyond their established diagnostic and prognostic value in TGCT—especially the clinical utility of circulating miR-371a-3p—miRNAs are increasingly recognized as upstream regulators of immune escape, checkpoint signaling, antigen presentation, inflammatory crosstalk, macrophage polarization, and extracellular-vesicle-mediated communication (12–18). Thus, miRNAs are not only biomarkers of disease activity, but also plausible mechanistic drivers of immunotherapy sensitivity or resistance.

In this review, we discuss how deregulated miRNAs may help explain the limited efficacy of current immunotherapeutic strategies in TGCT and how they could be exploited as biomarkers, therapeutic targets, or combinatorial tools to enhance anti-tumor immunity. By integrating the biology of TGCT, the current state of immunotherapy, and the immunoregulatory roles of specific miRNAs, we aim to outline a translational framework for more precise and effective immune-based strategies in this uniquely challenging disease.

This narrative review was based on a structured search of PubMed and Scopus databases, focusing on studies related to TGCT, microRNAs, and tumor immunology. Priority was given to TGCT-specific studies, while relevant findings from other malignancies were included when mechanistically informative. To improve conceptual clarity and avoid overinterpretation, the evidence discussed in this review is categorized into three levels (1): direct functional evidence in TGCT models, (2) biomarker-level evidence in TGCT, and (3) indirect evidence derived from other malignancies. This framework is applied throughout the manuscript to distinguish established findings from hypothesis-generating observations.

## 2 Testicular cancer, classification and management

It accounts for approximately 1–2% of all malignancies in men and ranks around the 20th most common cancers worldwide. Despite its relatively low overall incidence, it holds substantial clinical relevance as the most frequent solid tumors affecting men between 15 and 44 years of age, a period associated with high reproductive, professional and socioeconomic activity. Unique age distribution underscores the importance of timely diagnosis, optimal staging, and multidisciplinary management to minimize long-term morbidity and preserve quality of life. (1). Among testicular malignancies, testicular germ cell tumors (TGCTs) represent the vast majority. These arise from the malignant transformation of primordial germ cells (PGCs) or embryonic stem cells (ESCs) and are closely linked to early developmental processes. Disruptions in germ cell migration, differentiation, or maturation during embryogenesis are thought to contribute to tumor initiation (19).

TGCTs are broadly categorized into pure seminomas, which are generally less aggressive and represent approximately 52–56% of cases, and non-seminomatous germ cell tumors (NSGCTs), comprising 44–48% (1). NSGCTs are histologically heterogeneous and include embryonal carcinoma, yolk sac tumor, choriocarcinoma, teratoma, and mixed germ cell tumors. Importantly, any tumor containing both seminomatous and non-seminomatous elements is classified as an NSGCT, regardless of the relative proportion of each element. While this classification is fundamental for clinical decision-making, it does not fully capture the biological and immunological heterogeneity that characterizes TGCTs.

Clinically, testicular tumors most commonly present as a painless testicular mass, testicular enlargement, or as incidental findings on scrotal ultrasonography. Serum tumor markers (STMs)—including α-fetoprotein (AFP), β-human chorionic gonadotropin (β-hCG), and lactate dehydrogenase (LDH)—remain integral to diagnosis, staging, risk stratification, and post-treatment surveillance. These markers should be assessed prior to orchiectomy and re-evaluated postoperatively according to their biological half-lives. However, STMs have important limitations: only a minority of pure seminomas and approximately half of NSGCTs demonstrate elevated levels, and false-positive results may occur in non-malignant conditions or other cancers (20). These shortcomings highlight the need for novel biomarkers capable of reflecting tumor biology, immune dynamics, and treatment response more accurately.

### 2.1 Therapeutic management of testicular germ cell tumors

The management of TGCTs is guided by histology, clinical stage, and prognostic risk stratification and typically involves a multimodal approach integrating surgery, chemotherapy, and, in selected cases, radiotherapy. Radical inguinal orchiectomy remains the cornerstone of diagnosis and the standard initial therapeutic intervention for a suspicious testicular mass, providing definitive local control and essential histopathological information (3). For patients with seminoma, management options include active surveillance, adjuvant carboplatin, or radiotherapy based on individual risk factors. In contrast, NSGCTs often require a more individualized strategy incorporating surveillance, retroperitoneal lymph node dissection (RPLND), and/or cisplatin-based chemotherapy. Cisplatin-containing regimens, most commonly BEP (bleomycin, etoposide, cisplatin) or EP (etoposide, cisplatin) have dramatically improved outcomes and constitute the backbone of systemic therapy. The integration of surgery and chemotherapy in a multimodal framework has resulted in excellent survival rates, particularly in early-stage disease (21). Clinical stage TGCTs achieve 5-year overall survival rates approaching 100% when managed according to contemporary clinical guidelines. In metastatic disease, patients are stratified into good, intermediate, and poor prognostic categories based on the International Germ Cell Cancer Collaborative Group (IGCCCG) classification. Despite differences in survival across these categories, cure rates remain remarkably high, reflecting the intrinsic chemosensitivity of TGCTs (2). Nonetheless, despite high cure rates, 20–30% of patients develop refractory disease or experience a relapse after first-line treatment (3). Management of relapsed disease depends on timing and disease characteristics.

For early relapse or progressive disease, conventional-dose salvage chemotherapy (CDCT) regimens such as TIP (paclitaxel, ifosfamide, cisplatin) or VeIP (vinblastine, ifosfamide, cisplatin) are commonly employed, with TIP achieving durable remission rates of up to 63% in selected cohorts (22). Patients failing CDCT may be candidates for high-dose chemotherapy (HDCT) with autologous stem cell rescue, which can induce long-term remission in approximately 60% of relapsed metastatic cases (3, 4).

Late relapses occurring more than two years after initial treatment are preferentially managed with surgical salvage when feasible, often resulting in durable disease control. RPLND also plays a critical role in the management of residual chemoresistant masses or teratoma following systemic therapy (4).

### 2.2 Challenges of the current therapeutic management

Despite these advances, several clinical scenarios remain particularly challenging. Testicular stromal cell tumors, including Sertoli and Leydig cell tumors, are largely resistant to chemotherapy and radiotherapy and are associated with significantly poorer outcomes compared with TGCTs (23). Furthermore, long-term survivors of TGCT treatment face substantial late toxicities, including cardiovascular disease, secondary malignancies, metabolic complications, and infertility (4, 22). When HDCT fails, the prognosis is exceptionally poor, with survival often limited to a few months (24). These limitations underscore the urgent need for novel therapeutic strategies and improved biomarkers to better stratify patients and guide treatment.

## 3 Immunotherapy in cancer

Immunotherapy has become a “fourth pillar” of oncology alongside surgery, radiotherapy, and chemotherapy by shifting the therapeutic goal from directly killing tumor cells to mobilizing the patient’s own immune system to recognize and eradicate malignant cells (5, 6, 25). Rather than acting as a purely cytotoxic intervention, immunotherapy attempts to restart and sustain the tumor–immune cycle (26–28). The central promise is that, once appropriately activated and guided, adaptive immunity can generate specificity and memory, offering durable control of residual disease and prevention of recurrence and metastasis (29). Modern clinical immunotherapy comprises several complementary modalities that intervene at different points of tumor–immune interaction, some of which are:

- Immune checkpoint inhibitors (ICIs), monoclonal antibodies that block inhibitory receptor–ligand pathways, which tumors exploit to silence T cells—particularly PD-1 (nivomulab, pembrolizumab) PD-L1 (also known as B7-H1 or CD274) (atezolizumab, durvalumab, avelumab), and CTLA-4 (ipilimumab, tremelimumab), and in some contexts additional checkpoints such as LAG-3. ICIs aim to reinvigorate cytotoxic T lymphocytes (CTLs) and restore effective tumor killing (7, 30, 31).
- Adoptive cell therapy (ACT). Adoptive cell therapy (ACT) involves the modification of a patient’s own immune cells, which are expanded and/or engineered ex vivo and subsequently reinfused as “living drugs.” Chimeric antigen receptor T-cell (CAR-T) therapy exemplifies this approach by equipping T cells with synthetic receptors that recognize tumor surface antigens independently of major histocompatibility complex (MHC) presentation, enabling potent anti-tumor activity in selected hematologic and solid malignancies.
- Related strategies include tumor-infiltrating lymphocyte (TIL) therapy, engineered T-cell receptor (TCR) approaches, and emerging CAR-natural killer (CAR-NK) cell platforms. However, the application of these approaches in testicular germ cell tumors (TGCTs) remains largely unexplored. Challenges such as limited tumor-specific antigens, the immunoregulatory tumor microenvironment, and the influence of testicular immune privilege may restrict their effectiveness in this setting. Therefore, while ACT represents a promising immunotherapeutic modality in oncology, its relevance in TGCT should currently be considered exploratory and requires further disease-specific investigation (32–34).
- Therapeutic cancer vaccines. These stimulate a primary immune response and amplify tumor-directed adaptive immunity using tumor-associated antigens (TAAs) or patient-specific neoantigens, delivered through platforms such as dendritic cell–based vaccines or mRNA technologies, with the aim of eliciting durable T-cell memory (35–39).
- Oncolytic virus therapy. This therapy uses naturally occurring or engineered viruses that preferentially infect and lyse tumor cells, thereby releasing tumor antigens and inflammatory signals, including Damage-Associated Molecular Patterns (DAMPs), that can propagate systemic antitumor immunity, (T-VEC in melanoma) (40–42).
- Cytokines and other immunomodulators The administration of immune-stimulating signaling proteins like IL-2, IFNs, and pathway agonists such as STING agonists, can broadly stimulate immune activation, reshape the tumor microenvironment, and enhance effector-cell recruitment and function (42–45).

### 3.1 Success rate, pitfalls and challenges

Clinical benefit from immunotherapy is highly heterogeneous, varying according to tumor type, immune contexture, and the presence of predictive biomarkers. Melanoma is often cited as a paradigm for successful checkpoint blockade, with combined nivolumab and ipilimumab (anti–CTLA-4 plus anti–PD-1) achieving objective response rates (ORR) of approximately 50–60% and 5-year overall survival approaching 50% in advanced disease (7, 46).

Hematologic malignancies—particularly certain B-cell leukemias/lymphomas and Hodgkin lymphoma—have demonstrated some of the most pronounced responses to adoptive cell therapy (ACT), including CAR-T approaches, with high response or remission rates reported in selected patient populations (47). These outcomes highlight the potential of engineered immune strategies when tumor-specific antigens are well defined.

In contrast, across many common solid tumors, single-agent anti–PD-1/PD-L1 therapies typically yield more modest response rates, often in the range of 10–30%, and several malignancies remain relatively refractory in unselected populations (35, 38). For example, glioblastoma has shown limited clinical benefit from checkpoint inhibition, with large phase III trials failing to demonstrate significant survival advantages in unselected cohorts (48). Similarly, tumors characterized by low immunogenicity or “cold” immune phenotypes, such as ovarian cancer, generally exhibit modest response rates to immune checkpoint inhibitor (ICI) monotherapy (approximately 10–15%) (35).

Overall, a substantial proportion of patients—estimated to be between 40% and 60%—do not derive meaningful clinical benefit from currently available checkpoint inhibitors (18), underscoring the gap between the transformative potential of immunotherapy and its variable effectiveness at the population level. These limitations are driven by a combination of biological and clinical factors, including tumor-intrinsic features and the composition of the tumor microenvironment, which together shape responsiveness to immunotherapeutic interventions. Several biological and clinical barriers explain the limitations in immunotherapy efficacy and safety:

- Therapeutic resistance is central, appearing as *primary resistance* (no initial response, often associated with low neoantigen load, immune-desert phenotypes, or defective antigen presentation such as B2M-related mechanisms), and *acquired resistance* (escape after an initial response via antigen loss, pathway rewiring, or upregulation of alternative checkpoints such as TIM-3 or LAG-3) (49–51).
- The immunosuppressive tumor microenvironment (TME) has been described as a major physical and functional barrier: hypoxia, acidity, nutrient deprivation, dense stroma, and fibrosis can restrict immune-cell trafficking and blunt effector function (5). In parallel, tumors recruit immunosuppressive cell types such as: Tregs, MDSCs, and M2-polarized macrophages—that actively paralyze antitumor responses (30, 52–54).
- Exosomal interference. Tumors can secrete extracellular vesicles (EVs) carrying to neutralize T cells remotely, acting as decoys that dampen therapy efficacy at a distance (30, 52–55).
- On-Target Off-Tumor Toxicity: Over-activation of the immune system can cause autoimmune damage, also known as Immune-related adverse events (irAEs), which can involve skin, gut, lung, endocrine organs, heart, and other tissues (with severe colitis, pneumonitis, myocarditis noted among serious syndromes) (49, 56, 57). ACT adds distinct risks, especially cytokine release syndrome (CRS) and neurotoxicity in CAR-T settings. A related hazard is on-target off-tumor toxicity, in which target antigens are not truly tumor-exclusive and low-level expression in normal tissues can lead to critical organ damage (32, 58).

Finally, practical challenges shape real-world access and scalability: tumor heterogeneity (between patients and within the same tumor) complicates target selection and can drive incomplete eradication and relapse, often necessitating combination or personalized strategies (59). In addition, cost and manufacturing complexity are particularly prominent for individualized platforms such as CAR-T products and neoantigen vaccines, limiting availability even when efficacy is compelling (18, 60, 61). Also biomarker unreliability, especially the dynamic and imperfect predictive value of PD-L1 expression, contributes to both overtreatment and missed opportunities when used in isolation (62), which again, highlight the need for novel biomarkers capable of reflecting tumor biology, immune dynamics, and response to these treatments more accurately.

In the context of testicular germ cell tumors (TGCTs), these challenges may be further compounded by the unique immunological characteristics of the testis, including elements of immune privilege and a relatively low tumor mutational burden, which may limit the efficacy of conventional immunotherapeutic approaches.

## 4 Immunotherapy in testicular cancer

The current management of testicular germ cell tumors (TGCTs) has long been defined by the success of cisplatin-based chemotherapy, which cures approximately 80% of patients with metastatic disease (63). However, for the 15–20% of patients who develop platinum-refractory disease or relapse after high-dose chemotherapy (HDCT), the prognosis is often dismal, with survival frequently limited to a few months (64). Consequently, immunotherapy has emerged as a critical “last bullet” for these patients (65).

### 4.1 Rationale for immunotherapy in testicular cancer

The rationale for using immunotherapy in testicular germ cell tumors (TGCTs) is driven by the urgent clinical need to treat platinum-refractory disease and the unique immunological landscape of these tumors. Furthermore, because TGCTs predominantly affect young men, there is a strong motivation to find novel therapies with fewer long-term toxicities than standard chemotherapy regimens (66). The rationale is based on the next points:

- Expression of Immune Checkpoints (PD-L1/CTLA-4): A primary biological rationale for using immune checkpoint inhibitors (ICIs) is the frequent overexpression of immune checkpoints in TGCTs compared to normal tissue.
  - PD-L1 Expression: Programmed death-ligand 1 (PD-L1) is rarely expressed in normal testicular tissue but is frequently upregulated in TGCTs (67–70). Studies indicate PD-L1 expression in 73% of seminomas and 64% of non-seminomas (11). This high expression correlates with poor prognostic features, such as multiple metastatic sites and high serum tumor markers (9). Conversely, high levels of PD-L1 expression on tumor-infiltrating lymphocytes (TILs) have been associated with better progression-free survival (PFS) and overall survival (OS) (8). This data suggests that the PD-1/PD-L1 pathway is active in TGCTs and plays a role in immune tolerance and tumor dissemination (9).
  - CTLA-4: Cytotoxic T-lymphocyte-associated protein 4 (CTLA-4) is also highly expressed in TGCTs (89.7% of cases in one study), particularly in yolk sac tumors and choriocarcinomas, providing a rationale for targeting this checkpoint (71).
- Tumor Microenvironment and Infiltrating Lymphocytes. The testis is a prototypical immune-privileged organ, characterized by a tightly regulated microenvironment that limits immune activation to preserve germ cell integrity. This immune privilege is maintained through a combination of anatomical, cellular, and molecular mechanisms, including the blood–testis barrier, local secretion of immunosuppressive cytokines (e.g., TGF-β, IL-10), and active immunomodulation by Sertoli and Leydig cells. These cells contribute to antigen sequestration, suppression of T-cell activation, and induction of immune tolerance, thereby preventing autoimmune responses against germ cell-specific antigens (72–76). Beyond structural protection, the testicular microenvironment exhibits active immune regulation, including expression of immune checkpoint molecules such as PD-L1 and Fas ligand, which further contribute to T-cell apoptosis and immune suppression. Additionally, resident macrophages and dendritic cells in the testis tend to display tolerogenic phenotypes, favoring immune quiescence over activation. Together, these features create a highly specialized immunological niche that is fundamentally distinct from most other solid tissues. In the context of testicular germ cell tumors (TGCTs), this immune-privileged environment is partially disrupted but not fully abrogated. TGCTs arise from germ cells and therefore retain several intrinsic features of the testicular milieu, including low mutational burden, reduced neoantigen presentation, and persistent immunoregulatory signaling. Emerging evidence suggests that TGCTs exhibit a paradoxical immune phenotype, characterized by immune cell infiltration in some cases—particularly seminomas—yet limited effective anti-tumor immune responses. This apparent discrepancy may reflect the coexistence of immune activation and dominant immunosuppressive mechanisms within the tumor microenvironment. Importantly, TGCT subtypes differ in their immune composition and immunogenic potential. Seminomas are generally associated with higher levels of immune infiltration, including T lymphocytes and antigen-presenting cells, whereas non-seminomatous TGCTs (NSGCTs), particularly in the cisplatin-resistant setting, tend to exhibit a more immunosuppressive phenotype with reduced immune cell engagement. Mature teratomas, in contrast, are often characterized by low proliferative activity and limited immune recognition, further complicating therapeutic targeting. These unique immunological features have important therapeutic implications. Despite initial expectations, immune checkpoint inhibitors have demonstrated limited efficacy in TGCTs, even in heavily pretreated populations. This may be explained by the combination of low tumor mutational burden, restricted antigen presentation, and a persistently immunosuppressive microenvironment rooted in testicular immune privilege. As a result, TGCTs may represent a paradigm of “immune-cold” or immune-dysregulated tumors in which conventional immunotherapeutic strategies are insufficient to elicit durable responses. Within this context, regulatory mechanisms beyond classical checkpoint pathways are likely to play a critical role in shaping the tumor immune microenvironment. Among these, microRNAs have emerged as key modulators of immune cell differentiation, cytokine signaling, and checkpoint expression in multiple cancer types. Most of these findings derive from non-TGCT malignancies, the integration of miRNA-mediated regulation into this unique immune landscape provides a compelling framework to better understand immune escape and to identify novel strategies for therapeutic modulation. Taken together, the immune microenvironment of TGCTs reflects a complex interplay between residual immune privilege and tumor-driven immune evasion. A deeper understanding of this balance is essential for the development of effective immunotherapeutic approaches and provides a strong biological rationale for exploring miRNA-based regulatory mechanisms in this disease. In spite of that, the testis and TGCTs exhibit complex immune interactions:
  - Infiltration: TGCTs, particularly seminomas, are frequently characterized by a vigorous inflammatory infiltrate of activated CD8+ and CD4+ T cells (74). Studies have shown that patients with abundant PD-L1-positive TILs in the tumor microenvironment have significantly better outcomes (75).
  - Immune Surveillance: The presence of TILs and spontaneous CD4+ and CD8+ T-cell responses against cancer-testis antigens (CTAg) suggests that the patient’s immune system attempts to recognize the tumor (76). However, the tumor microenvironment (TME) is often immunosuppressive, characterized by regulatory T cells (Tregs) and macrophages that inhibit effective anti-tumor immunity (77). Immunotherapy aims to reverse this suppression and reinvigorate the existing T-cell response (76).
- Genomic and Epigenetic Factors.
  - Microsatellite Instability (MSI): While rare, MSI-high status and mismatch repair (MMR) deficiency are more common in cisplatin-resistant TGCTs than in chemo-naive tumors (78). These features are strong predictors of response to immunotherapy in other solid tumors, providing a rationale for testing ICIs in this specific subset of refractory patients (9).
  - Hypomethylation and Viral Mimicry: Seminomas are characterized by global DNA hypomethylation (66). This state correlates with the abundance of infiltrated CD8+ cells and also with the re-expression of human endogenous retroviruses (HERV), which in turn, triggers type-I interferon signaling. This “viral mimicry” renders the tumor more immunogenic, suggesting a potential synergy between hypomethylating agents and immunotherapy (79).
- Chemotherapy-Induced Immunogenicity.

There is a rationale for combining immunotherapy with chemotherapy. Cytotoxic agents like cisplatin can induce immunogenic cell death, which may broaden the range of tumor antigens released and stimulate the production of Type I interferons, potentially creating a more favorable environment for immunotherapy to work. Furthermore, chemotherapy can downregulate immunosuppressive checkpoint molecules and upregulate MHC class I molecules, which are critical for antigen presentation (77, 79).

### 4.2 Immunotherapy approaches in testicular cancer: what’s been failed and what’s been succeeded

Immunotherapy approaches in TGCTs—specifically for platinum-refractory disease—have yielded mixed results. Despite strong biological rationales, the clinical results for standard checkpoint inhibitors have been largely disappointing. Still, there are some successful examples of novel cellular therapies and biomarker-driven cases.

#### 4.2.1 What has succeeded: targeted cellular therapies and biomarker-selected cases

Success has been observed when moving beyond generic checkpoint inhibition toward highly specific targets or genetically distinct tumor subsets:

• **Chimeric Antigen Receptor (CAR) T-cells engineered to target Claudin-6 (CLDN6),**

Specific surface antigens on testicular germ cell tumors (TGCTs) provide a rationale for cellular immunotherapies like CAR T-cell therapy, going beyond checkpoint inhibition. Claudin-6 (CLDN6) is an oncofetal antigen that is silenced in healthy adult tissues but highly expressed in approximately 93% of TGCTs. This strict tumor-specificity makes CLDN6 an ideal target for CAR-T therapy to minimize off-target toxicity. Early trials targeting CLDN6, combined with an mRNA vaccine (CARVac) to boost T-cell persistence, have shown encouraging objective response rates (57%) in refractory GCT patients (77). Recently, an ongoing clinical trial (Phase I) testing a CAR-T cell (BNT211) against CLDN6 as monotherapy and in combination with CLDN6-encoding CAR-T-cell-amplifying RNA vaccine (CARVac) in a GCT cohort with CLDN6-positive solid tumors was launched. Thirteen patients showed an objective response rate (ORR) of 57% and a disease control rate of 85%. One patient achieved a complete response. CAR-T cells persisted for over 100 days (11, 77).

• **Tumor Mutational Burden (TMB) and Microsatellite Instability-High (MSI-H)**.

There are still some few case reports where a very small subset of patients responded exceptionally well to ICIs. For example, a patient with chemotherapy-refractory GCT and MSI-High status achieved a rapid and sustained response to pembrolizumab (80). Another patient with high TMB had a durable partial response to nivolumab lasting 90 weeks (8).

• **DDR Gene Mutations**.

Another example of success is a case where a patient with metastatic mixed GCT harboring co-mutations in DNA damage-repair (DDR) genes (BRCA2, MSH6, PMS2) achieved a long-term response (>28 months) to the PD-1 inhibitor camrelizumab (81).

• **Antibody-Drug Conjugates (ADCs): Moderate Success**.

Brentuximab vedotin (anti-CD30) represents an example of a targeted antibody–drug conjugate that has been explored in testicular germ cell tumors (TGCTs), particularly in embryonal carcinomas and a subset of seminomas characterized by CD30 expression. This agent delivers a cytotoxic payload directly to CD30-expressing cells, providing a mechanism for selective tumor targeting.

Early clinical observations, including small case series, reported partial and complete responses, with at least one durable complete remission exceeding four years. However, subsequent prospective evaluation in a phase II trial demonstrated more limited activity, with objective responses observed in only 2 of 7 patients, including one durable complete remission, and no responses in an additional cohort of 18 patients (9, 11).

These findings suggest that the clinical benefit of brentuximab vedotin in TGCT may be restricted to a small subset of patients with CD30-positive tumors. The variable efficacy may reflect both biological and clinical factors, including the predominance of CD30 expression in specific histological subtypes such as embryonal carcinoma, as well as the potential loss of CD30 expression following chemotherapy, which may limit therapeutic effectiveness in later lines of treatment (82).

Overall, while this approach illustrates the potential of antigen-directed therapies in TGCT, its role remains limited and should be considered investigational, particularly outside of carefully selected patient populations or combination strategies.

#### 4.2.2 What has failed: immune checkpoint inhibitors in unselected patients

The most extensively tested approach has been the use of inhibitors targeting PD-1, PD-L1, and CTLA-4. Despite high levels of PD-L1 expression in TGCTs, (up to 73% in seminomas) and high levels of tumor-infiltrating lymphocytes (TILs), clinical trials using ICIs as monotherapy have been largely disappointing.

- Pembrolizumab (Anti-PD-1): Two separate Phase II trials showed no clinically meaningful single-agent activity.
  - Adra et al. reported zero objective responses among 12 patients with refractory GCT. Only two patients achieved stable disease, and the trial was closed early for futility (63).
  - Tsimberidou et al. reported similar results in 12 patients, with no objective responses and a median progression-free survival of only 2.4 months (10).
- Avelumab (Anti-PD-L1): A Phase II trial in multiple relapsed/refractory patients was terminated early because none of the eight heavily pretreated patients achieved the primary endpoint of 12-week progression-free survival; all patients progressed (9).
- Durvalumab (Anti-PD-L1) +/- Tremelimumab (Anti-CTLA-4): In the APACHE trial, the arm receiving durvalumab monotherapy was closed prematurely because 72.7% of patients experienced hyperprogression (rapid acceleration of disease) (9).
- Nivolumab (Anti-PD-L1): A Phase II trial of 17 patients showed only one partial response and three stable diseases. The response rate was considered low (8).

##### 4.2.2.1 Why did these fail?

Several biological and clinical factors may contribute to the limited efficacy of immunotherapy in testicular germ cell tumors (TGCTs).

• Low tumor mutational burden (TMB). TGCTs are generally characterized by a relatively low TMB compared to highly immunogenic tumors such as melanoma (79). Although isolated cases with higher mutational burden or microsatellite instability (MSI) have been reported, these represent a small subset. Lower TMB is typically associated with reduced neoantigen load, which may limit immune recognition and the effectiveness of immune checkpoint blockade (11).

• Immune privilege and tissue-specific homeostasis. The testis is an immunologically privileged organ (79). Under physiological conditions, local immune tolerance mechanisms protect developing germ cells from autoimmune attack. These include the blood–testis barrier and the expression of immunoregulatory molecules such as PD-L1 and FAS ligand (FASL) by Sertoli and Leydig cells. Elements of this tolerogenic microenvironment may persist in TGCTs and contribute to immune evasion, potentially limiting the penetration and/or activity of systemic immunotherapies (83).

• Limited predictive value of current biomarkers. The role of PD-L1 as a predictive biomarker in TGCT remains inconsistent. While PD-L1 expression can be observed in a subset of tumors, it does not reliably correlate with response to checkpoint inhibition. Notably, PD-L1 expression on tumor cells has been associated with poorer prognosis, whereas expression on tumor-infiltrating lymphocytes (TILs) may correlate with improved outcomes, suggesting a complex and context-dependent relationship that limits its clinical utility as a predictive marker (78, 84).

Together, these factors highlight the complexity of the TGCT immune microenvironment and underscore the need for alternative or combinatorial strategies to enhance immunotherapy responsiveness.

#### 4.2.3 Why miRNAs are a logical next step in TGCT immunotherapy

The limited activity of single-agent ICIs in unselected patients with TGCT, despite frequent PD-L1 expression, suggests that static checkpoint assessment alone may not fully capture the biological determinants of immune responsiveness in this disease. In this context, miRNAs are attractive because they may function as dynamic biomarkers and, potentially, as therapeutic modulators. On the biomarker side, TGCT already provides one of the strongest examples of clinical miRNA utility in solid tumors: circulating miR-371a-3p, which consistently outperforms conventional serum markers for detecting active malignant TGCT (except pure teratoma), correlates with disease burden, and tracks treatment response. These observations suggest that miRNA measurements can reflect real-time tumor biology in TGCT and may provide complementary information to static biomarkers such as PD-L1 immunohistochemistry (12–15).

Beyond their role as biomarkers, miRNAs are increasingly recognized as potential regulators of pathways relevant to immunotherapy. In multiple cancer types, they have been shown to influence several layers of tumor immune escape, including PD-L1 expression, antigen-presentation programs, cytokine signaling, macrophage polarization, T-cell dysfunction, and extracellular vesicle–mediated crosstalk. In this context, miRNAs may act as upstream modulators of pathways that contribute to determining whether tumors exhibit “hot” or “cold” immune phenotypes, as well as sensitivity or resistance to immunotherapy. However, most of these observations derive from non-TGCT malignancies, and their applicability to TGCT remains to be fully defined. This conceptual framework may be particularly relevant in TGCT, given its unique biological features, including elements of immune privilege, relatively low tumor mutational burden, and a complex tumor microenvironment that may collectively limit responsiveness to checkpoint blockade. Accordingly, a miRNA-based approach could help identify biologically distinct TGCT subsets and, in selected contexts, suggest candidate pathways or targets that warrant further investigation before therapeutic application, including strategies based on miRNA mimics or anti-miR/antagomir platforms (16–18).

MicroRNAs have emerged as important post-transcriptional regulators of immune checkpoint pathways across multiple cancer types. Among these, miR-34a has been extensively studied for its role in modulating programmed death-ligand 1 (PD-L1) expression. In several solid tumors, including melanoma and non-small cell lung cancer, miR-34a has been shown to directly target PD-L1 mRNA, leading to reduced protein expression and enhanced T-cell–mediated anti-tumor responses. These findings have positioned miR-34a as a potential link between tumor-intrinsic signaling and adaptive immune evasion. However, the relevance of this regulatory axis in testicular germ cell tumors (TGCTs) remains largely undefined. To date, there is limited direct functional evidence demonstrating that miR-34a modulates PD-L1 expression or immune responses in TGCT-specific models. Available data in TGCT are primarily descriptive and do not yet establish a causal relationship between miRNA expression and immune checkpoint regulation. On the other hand, the therapeutic potential is not merely hypothetical. In oncology, the first-in-human studies of MRX34, a liposomal miR-34a mimic, provided proof-of-concept that systemic miRNA replacement is pharmacologically feasible in cancer patients, even though clinical development was ultimately limited by serious immune-mediated toxicities. However, the experience is highly instructive for TGCT because it supports the feasibility of miRNA-directed therapy, while also emphasizing that delivery platform, patient selection, immune context, and rational combinations are also critical if miRNA therapeutics are to be integrated with immunotherapy safely and effectively. Therefore, miRNAs offer a biologically coherent bridge between the current failure of conventional biomarkers and the need for more precise, mechanism-based immunotherapy strategies in TGCT (10, 85, 86). As such, extrapolation from other malignancies should be interpreted with caution. From a biological perspective, the applicability of miR-34a–mediated checkpoint regulation in TGCT may be influenced by the unique features of the testicular tumor microenvironment. TGCTs typically exhibit a low tumor mutational burden and a partially preserved immune-privileged milieu, both of which may limit the effectiveness of checkpoint-based immune activation. In this context, even if miR-34a were to modulate PD-L1 expression, the downstream impact on anti-tumor immunity could differ substantially from that observed in more immunogenic tumors.

Despite these limitations, the miR-34a/PD-L1 axis provides a useful conceptual framework for understanding how miRNA-mediated regulation could influence immune escape mechanisms in TGCT. Rather than representing an established therapeutic target, this pathway should currently be viewed as a hypothesis-generating model that warrants further investigation in disease-specific systems. Future studies incorporating TGCT cell lines, patient-derived samples, and functional immune assays will be necessary to determine whether modulation of miR-34a can meaningfully alter immune responsiveness in this setting. More broadly, this example highlights a recurring challenge in the field: while miRNA-mediated immune regulation is well supported in other cancer types, its direct translation to TGCT remains limited by a lack of functional validation. Addressing this gap will be essential for advancing miRNA-based immunomodulatory strategies from conceptual models to clinically relevant applications in TGCT.

## 5 Deregulated microRNAs in testicular cancer and treatment response

### 5.1 Diagnostic and prognostic role

To conceptualize the function of microRNAs, one might imagine the genes within a cell as light bulbs whose brightness determines the level of protein produced. In this analogy, miRNAs act as the dimmer switches, fine-tuning the intensity of gene expression rather than turning the lights fully on or off. Biologically, miRNAs are short non-coding RNAs (ncRNAs) that regulate gene expression at the post-transcriptional level. Unlike messenger RNAs that encode protein sequences, miRNAs do not direct protein synthesis; instead, they modulate it by binding to target mRNAs and influencing their stability or translational efficiency. Through this precise regulatory mechanism, miRNAs exert substantial control over cellular function and play a pivotal role in maintaining normal physiology as well as contributing to disease when dysregulated. Dysregulation at any step of miRNA regulation can lead to aberrant gene expression patterns that promote the initiation and progression of several malignancies. Within the context of TGCTs, miRNAs play a critical role in tumor initiation, cellular proliferation, and the maintenance of *stemness* or self-renewal capacity. By regulating the post-transcriptional expression of genes involved in pluripotency, differentiation, and cell-cycle control, miRNAs help preserve biological characteristics of primordial germ cells and embryonic stem cells, central features to the development and progression of TGCTs (87).

The first evidence of miRNA involvement in TGCTs demonstrated that the miR-371~373 cluster, located on chromosome 19q13, is markedly overexpressed in adult testicular cancer. Subsequent functional studies suggested that this cluster may act as a potential oncogenic driver, in part through the inhibition of LATS2 (Large Tumor Suppressor Kinase 2), a key component of the Hippo signaling pathway (88). Particularly, the miR-371–373 cluster has been identified as an oncogenic driver in TGCTs, as it is able to counteract the tumor-suppressive effects of p53 by blocking Ras-induced cellular senescence. The molecular basis of this upregulation has been linked to a regulatory feedback loop between the miR-371–373 cluster and the Wnt/β-catenin signaling pathway. Notably, higher expression levels of this miRNA cluster have been observed in stem cells compared with differentiated cells. Given the central role of Wnt signaling in the regulation of cellular stemness, these findings suggest that the miR-371–373 cluster plays a key role in maintaining the undifferentiated, stem-like phenotype of TGCT cells (89).

Multiple studies have demonstrated that circulating miRNAs can outperform conventional serum tumor markers in the detection of active disease, longitudinal monitoring, and relapse prediction in TGCT (90). In particular, members of the miR-302/367 and miR-371–373 clusters have shown high diagnostic accuracy for primary tumors and have been associated with improved sensitivity in disease monitoring and early relapse detection compared with traditional markers (91).

Among these, miR-371a-3p has emerged as one of the most promising non-invasive biomarkers in TGCT, with consistent evidence supporting its clinical utility for diagnosis and treatment monitoring, although its performance remains limited in specific contexts such as pure teratoma.

### 5.2 miRNA expression profiling and clinical significance among TGCT subtypes

**Figure 1:**
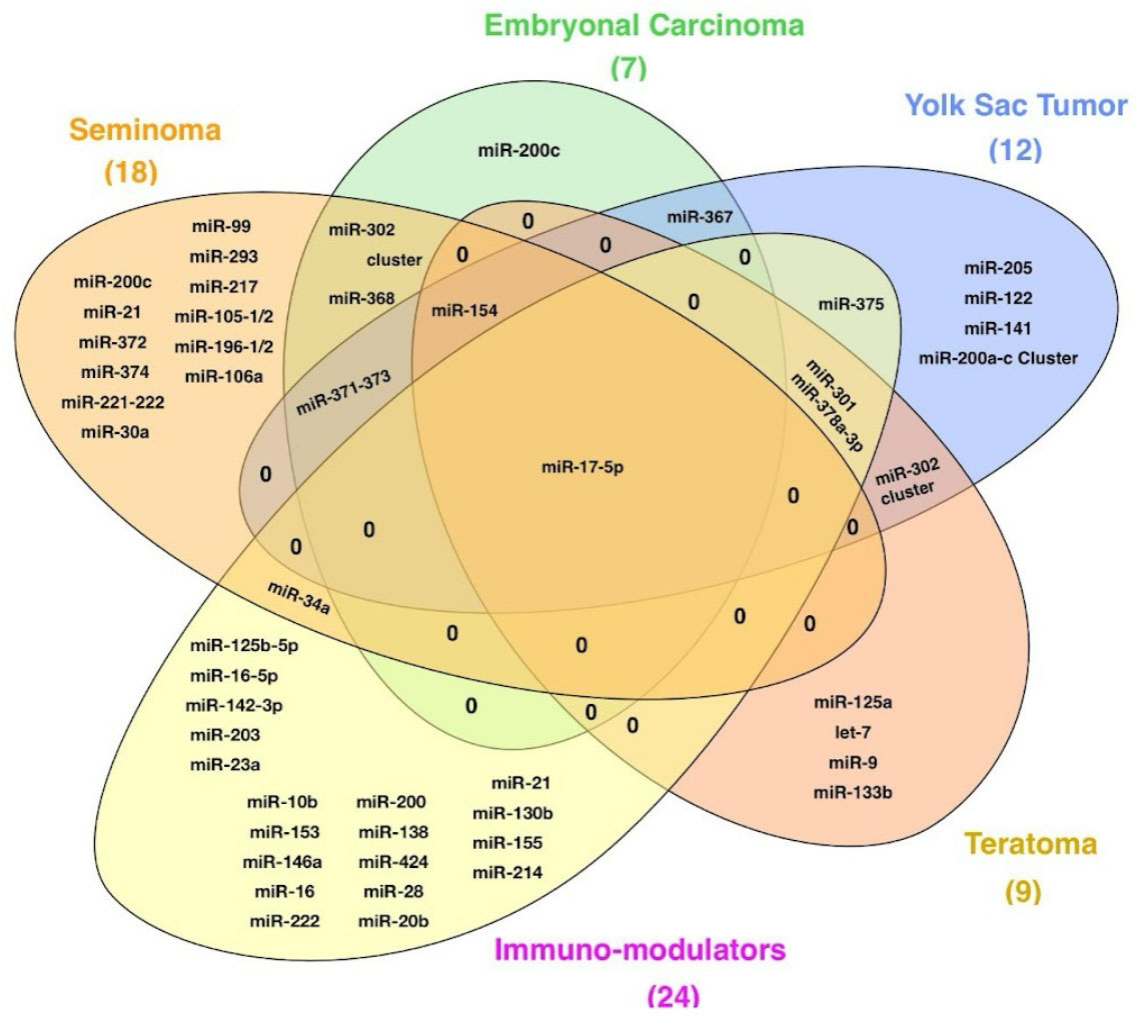
Deregulated miRNAs across TGCT subtypes and overlap with immunoregulatory candidates. Deregulated miRNAs reported in TGCT subtypes (seminoma, embryonal carcinoma, yolk sac tumor, and teratoma), including shared and subtype-associated miRNAs. The overlap includes miRNAs that are also implicated in immune regulation in other malignancies (group of immune-modulators), representing candidate modulators of the tumor immune microenvironment and potential contributors to immunotherapy response in TGCT. (Ref 50–200).

miRNA profiling also serves as a powerful tool for distinguishing the major histological variants of TGCTs (92). In seminoma, for instance, a distinct miRNA expression signature has been identified when compared with normal testicular tissue: miR-221, miR-222, miR-372, and miR-374 are consistently upregulated, whereas others, including miR-30a, miR-34a, miR-99, miR-105-1/2, miR-106a, miR-293, miR-217, miR-196-1, and miR-196-2, are markedly downregulated (93). Notably, the miR-302 cluster, normally expressed in human embryonic stem cells, is highly expressed in embryonal carcinoma and seminoma but becomes downregulated as embryonal carcinoma differentiates into teratoma. A similar differentiation-dependent reduction is observed for miR-17-5p and miR-154. In contrast, miR-301 shows predominant expression in more differentiated subtypes, such as spermatocytic seminomas, yolk sac tumors, and teratomas, while being absent in embryonic stem cells and embryonal carcinoma. These and other miRNAs are shown in **Figure 1**.

### 5.3 From diagnosis to risk stratification in testicular germ cell tumors: the case of the cluster miR-371~373

In seminoma, miR-371a-3p is one of the most clinically relevant circulating miRNAs, showing higher expression levels with tumor stage, while declining after orchiectomy or chemotherapy, which supports its diagnostic and disease-monitoring value (94–96). Its incorporation into the diagnostic pipeline may also facilitate earlier detection, since overexpression has been identified in a substantial proportion of Germ Cell Neoplasia *In Situ* (GCNIS). In addition, it may also assist in preoperative decision-making, specially when a testis-sparing surgery is being considered (97, 98). Although specificity of miR-371a-3p is high in seminoma, sensitivity may decrease in very small lesions, indicating some dependence on tumor burden (99, 100). From a prognostic perspective, higher serum miR-371a-3p levels have been associated with seminoma relapse, suggesting utility for detecting early recurrence (101). However, while elevated pretreatment levels correlate with adverse disease features in the overall TGCT population and particularly in non-seminoma, the prognostic benefit appears limited in the seminoma group and patients with negative serum tumor markers. Therefore, in these subgroups the disease response could be evaluated only with imaging studies (102).

In non-seminomatous TGCTs, including embryonal carcinoma and yolk sac tumor, the miR-371~373 cluster is also strongly expressed (**Figure 1**) and contributes to tumorigenesis by functionally impairing the p53 pathway, reinforcing its biological and diagnostic relevance (96). miR-371a-3p shows high diagnostic accuracy in non-seminoma, with 95% sensitivity and 96.1% specificity in a large prospective multicenter study, and retains good performance even in small non-seminomatous lesions, making it particularly useful when conventional serum tumor markers are negative or inconclusive (99, 100). Additional studies confirmed high specificity and positive predictive value, although sensitivity and negative predictive value vary across methodologies and follow-up settings (103, 104). In patients with clinical stage IIA and IIB (CSI–IIA/B) and negative serum tumor markers undergoing RPLND, elevated plasma miR-371a-3p accurately predicted non-teratomatous metastases, with an AUC of 0.965, 100% sensitivity, and 92% specificity (105). Within teratoma, expression remains biologically heterogeneous: miR-371 has been reported as absent in persistent teratoma and fibrosis (106), yet higher miR-371a-3p expression has been described in postpubertal teratomas and GCNIS-related TGCTs (107), and miR-371 has been also detected in cystic fluid from pure teratoma despite negative serum findings (108). In post-chemotherapy residual disease, combined miR-371/miR-375 analysis improved discrimination between benign tissue and residual malignant tumor, while miR-375 alone identified teratoma with 78% sensitivity, 80% specificity, and an AUC of 0.7 (14). Prognostically, higher pretreatment miR-371a-3p levels were associated with retroperitoneal nodal involvement, distant metastasis, and advanced clinical stage, whereas undetectable pre-therapy levels predicted better PFS and OS in non-seminomatous tumors and in advanced-stage disease (1057).

Taken together, these findings establish miR-371a-3p as a robust biomarker for TGCT detection, disease burden assessment, and risk stratification across histological subtypes. At the same time, the heterogeneity observed in teratoma and residual disease underscores that miRNA biology in TGCT is more complex than a purely diagnostic framework. This provides a strong rationale for examining how miRNA networks also influence therapeutic response, treatment resistance, and chemosensitivity in TGCT.

### 5.4 Therapy response and chemoresistance

Prospective trials have demonstrated miRNAs utility in monitoring chemotherapy response, where levels change significantly during treatment and predict the presence of viable tumor after post-chemotherapy retroperitoneal lymph node dissection. Interestingly, a “microRNA switch” has been associated in testicular germ cell tumors with tumor differentiation. The miR-371–373 cluster is upregulated in malignant subtypes, promoting a pro-apoptotic response to cisplatin. In contrast, mature teratomas show downregulation of this cluster and upregulation of miR-885-5p, leading to cell cycle arrest and contributing to cisplatin resistance (109). Several miRNAs influence cisplatin sensitivity and resistance. Patients with p53-positive TGCTs and upregulated miR-371–373 cluster appear to be more sensitive to chemotherapy and radiation, reflecting the differentiation-dependent microRNA switch described in germ cell tumors (107, 110). Additionally, whereas miR-302a and miR-383 enhance cisplatin sensitivity by promoting apoptosis and inducing G2/M cell-cycle arrest, the miR-371–373 cluster, has been associated with drug resistance and poor treatment response. Additional resistance-related miRNAs include miR-375, miR-378a-3p, and miR-20b-5p, whereas tumor-suppressor miRNAs such as miR-34a inhibit proliferation and promote apoptosis (73, 93, 111).

## 6 Immunotherapy response modulated by miRNAs

MicroRNAs are increasingly recognized as post-transcriptional regulators that may influence responses to cancer immunotherapy by modulating gene expression in both tumor cells and immune cell populations. In multiple malignancies, miRNAs have been shown to affect processes that contribute to the balance between effective anti-tumor immunity and immune escape.

In the context of immunotherapy, these mechanisms include: (i) regulation of immune checkpoint pathways, (ii) modulation of the tumor immune microenvironment (TME) through effects on differentiation, recruitment, polarization, and functional states of key immune populations, and (iii) intercellular communication via extracellular vesicles (EVs)/exosomes that may facilitate the spread of resistance-associated signals across the TME. However, it is important to note that most of these observations derive from non-TGCT models, and their relevance in TGCT remains to be fully established.

### 6.1 Modulation of immune checkpoint molecules

One of the most studied mechanisms through which microRNAs may influence anti-tumor immunity is the regulation of immune checkpoint pathways, particularly the PD-1/PD-L1 axis and other inhibitory receptors associated with T-cell exhaustion. In multiple cancer types, several miRNAs have been shown to modulate checkpoint expression either directly or through upstream regulatory networks.

For example, miR-34a and members of the miR-200 family have been reported to reduce PD-L1 expression in different tumor models (35, 112), while miR-138 has been shown to target multiple checkpoint pathways, including PD-1 and CTLA-4, and to modulate regulatory T-cell–associated markers such as FOXP3 (29, 113, 114). Similarly, miR-424 has been associated with reduced PD-L1 expression and modulation of CD80/CTLA-4 interactions, with downstream effects on CD8+ T-cell function (115, 116). In addition, miR-28 has been implicated in the regulation of T-cell exhaustion by influencing the expression of inhibitory receptors such as PD-1, TIM-3, and BTLA (117, 118).

Conversely, certain miRNAs, including miR-20b, miR-21, and miR-130b, have been reported in other malignancies to promote immune evasion by indirectly upregulating checkpoint molecules through PTEN suppression and activation of PI3K signaling pathways, leading to increased PD-L1 expression (119).

However, it is important to emphasize that most of these findings derive from non-TGCT models, and their functional relevance in TGCT remains to be established. Accordingly, therapeutic strategies based on restoring or inhibiting specific miRNAs should currently be considered hypothesis-generating and require validation in disease-specific systems.

### 6.2 Modulation of tumor microenvironment

The TME is the ecosystem that surrounds and interacts with malignant cells. In solid tumors, it is typically described as a dynamic network of cancer cells, stromal elements, and diverse immune populations that co-evolve with the tumor and collectively influence progression, metastasis, and therapeutic response (120). In addition to malignant cells, key TME components include stromal populations such as cancer-associated fibroblasts (CAFs), which can create both physical and biochemical barriers to immune infiltration, as well as multiple immune cell subsets, including T cells (CD8^+^ cytotoxic, CD4^+^ helper, and regulatory T cells), B cells, natural killer (NK) cells, dendritic cells (DCs), and myeloid populations such as tumor-associated macrophages (TAMs), tumor-associated neutrophils (TANs), and myeloid-derived suppressor cells (MDSCs) (120, 121).

Within this context, miRNAs are increasingly recognized as potential regulators of the cellular and molecular interactions that shape the TME. In multiple cancer types, miRNAs have been shown to influence immune cell differentiation, recruitment, polarization, and functional states, thereby contributing to the balance between effective anti-tumor immunity and immune escape. However, it is important to note that most of these observations derive from non-TGCT malignancies, and their relevance in TGCT remains to be fully defined. One of the most studied mechanisms involves the expansion and functional reinforcement of MDSCs, a heterogeneous population of myeloid cells associated with chronic inflammation that suppress T-cell activity and promote an immunosuppressive cytokine milieu (122). MDSCs exert their effects through multiple overlapping pathways, including metabolic suppression and the production of reactive oxygen and nitrogen species as well as immunomodulatory cytokines (123, 124). In this context, specific miRNAs have been implicated in the regulation of myeloid cell function. For example, a melanoma-associated cluster of tumor-derived miRNAs (including miR-146a/b, miR-155, miR-125b, miR-100, let-7e, miR-125a, and miR-99b) has been associated with myeloid reprogramming and resistance to immune checkpoint inhibitors (125). Mechanistically, miR-21 and miR-155 have been linked to immunosuppressive signaling through activation of STAT3-related pathways via targeting of SHIP-1 and PTEN, thereby promoting MDSC expansion in other malignancies (126, 127).

miRNAs have also been shown to influence T-cell functionality, including differentiation, metabolic programming, cytotoxic activity, and the development of exhaustion phenotypes. For instance, miR-155 has been reported to enhance T-cell effector function and cytokine production in preclinical models (114), whereas its loss has been associated with impaired anti-tumor immunity that may be partially rescued by checkpoint blockade (128). Conversely, tumor-derived miRNAs can contribute to immune suppression through intercellular transfer mechanisms. For example, miR-214 has been described as promoting regulatory T-cell (Treg) expansion via PTEN downregulation, thereby dampening anti-tumor immune responses (118, 129).

The TME’s macrophage axis is also strongly miRNA-driven. Exosomal miRNAs often mediate macrophage polarization (M2/M1), which dictates antigen presentation quality, cytokine production, and T-cell recruitment. MiRNAs can shift macrophages towards the pro-tumoral (M2-like) phenotype, often termed Tumor-Associated Macrophages (TAMs), hindering anti-tumor responses. One example is miR-21, derived from tumor cells, which promotes M2 polarization and immunosuppression (through PD-L1 expression) while blocking JAK2/STAT1 and engaging PTEN/PI3K/AKT signaling (130, 131). Secreted through tumor exosomes, miR-21 engages Toll-like receptors (TLR7/8) signaling to induce pro-metastatic inflammatory responses in macrophages (132). MiR-125b-5p is another consistent TAM-linked miRNA, described as promoting M2 phenotype features in melanoma (via exosomal delivery) by inhibiting Lysosomal Acid Lipase A (LIPA) (133, 134). While tumor-derived exosomes regulate the phenotype and function of immune cells that can be beneficial for tumor evasion and immune destruction, immunocyte-released exosomes regulate the anti-tumor immune response, influencing various biological functions of both host and recipient cells. For instance, M1 macrophage-derived exosomes transferring miR-16-5p to gastric cancer cells triggers T cell immune response by reducing the expression of PD-L1, which inhibits gastric cancer (GC) progression (29). Alternatively, *in vitro* studies have shown that miR-34a might be immunorestorative in part by promoting M1 polarization (via IL-6R depletion) while also directly targeting PD-L1 (via P53 pathway activation), placing it at the intersection of checkpoint repression and macrophage repolarization (135).

Dendritic cells (DCs) and NK cells are also subject to miRNA-mediated regulation, often through extracellular vesicle–dependent communication. Effective anti-tumor immunity depends on proper DC maturation and antigen presentation, processes that can be modulated by miRNAs. For example, miR-142-3p transferred from regulatory T cells to DCs has been associated with a tolerogenic phenotype characterized by increased IL-10 and reduced IL-6 production (136). Tumor-derived exosomal miR-203 has been reported to impair DC function by targeting TLR4, thereby reducing inflammatory cytokine production (137). Similarly, NK cell activity can be suppressed by tumor-derived miRNAs; hypoxia-associated miR-23a has been shown to reduce NK cell cytotoxic function and cytokine production in preclinical models (138). Additionally, tumor-intrinsic miRNAs such as miR-34a/c and miR-10b have been implicated in the downregulation of ligands required for NK cell recognition, contributing to immune evasion (139, 140).

Finally, cancer-associated fibroblasts (CAFs) are increasingly recognized as active regulators of immune exclusion and therapeutic resistance. Through extracellular matrix remodeling and cytokine signaling, CAFs can restrict immune cell infiltration and limit effective immune responses. miRNAs have been implicated in CAF activation and function; for example, miR-21 has been associated with fibroblast activation, extracellular matrix remodeling, and the promotion of immunosuppressive signaling pathways in various tumor types (141, 142).

Taken together, these observations suggest that miRNAs may represent an additional regulatory layer within the TME that contributes to immune evasion and variability in immunotherapy response. However, further functional validation in TGCT-specific models is required to determine the extent to which these mechanisms are operative in this disease.

### 6.3 MicroRNA-based strategies to enhance cancer immunotherapy

Cancer immunotherapy has transformed the management of multiple malignancies; however, durable clinical benefit remains limited by primary and acquired resistance, heterogeneity in tumor immunogenicity, and treatment-related immune toxicities. MicroRNAs have emerged as potential modulators of anti-tumor immunity, as they can influence multiple interconnected pathways, including checkpoint signaling (e.g., PD-1/PD-L1), antigen presentation, cytokine networks, and the differentiation and function of key immune and stromal populations.

Importantly, miRNAs may act not only within individual cells but also across the tumor microenvironment through extracellular vesicle (EV)/exosome-mediated transfer, thereby contributing to the propagation of immunosuppressive or immunostimulatory signals. These properties suggest that miRNAs could represent a versatile layer of immune regulation.

From a translational perspective, miRNA-based strategies—including the use of mimics to restore tumor-suppressive or immune-activating miRNAs, or inhibitors (antagomiRs) to target oncogenic miRNAs—have shown promise in preclinical models. In addition, their potential integration into cellular therapies or delivery platforms aimed at modulating the tumor microenvironment represents an area of active investigation.

Taken together, these approaches may provide a conceptual framework to enhance existing immunotherapeutic strategies, including checkpoint blockade, adoptive cell therapies such as CAR-T, and vaccine-based approaches. However, most of the current evidence derives from non-TGCT models, and further validation in disease-specific systems will be required to determine their clinical applicability, particularly in TGCT.

#### 6.3.1 Exosomes/extracellular vesicles: reprogramming the TME to favor immunotherapy

Extracellular vesicle (EV)–associated miRNAs have been implicated in intercellular communication within the tumor microenvironment, where they may contribute to immunosuppressive signaling. In several tumor models, EV-derived miRNAs have been associated with macrophage polarization toward M2-like phenotypes, expansion of regulatory T cells (Tregs), and modulation of PD-1/PD-L1 signaling pathways. Conversely, preclinical studies suggest that modulation of EV-mediated miRNA transfer—either by inhibiting tumor-derived signals or by delivering immunostimulatory miRNAs—may influence the immune landscape of the tumor microenvironment. These approaches have been associated with changes such as reduced PD-L1 expression, enhanced antigen presentation, increased pro-inflammatory cytokine signaling, and decreased suppressive immune cell populations, which may collectively support responsiveness to immune checkpoint inhibitors.

An illustrative example is miR-16-5p, which has been reported in preclinical models to be delivered via M1 macrophage–derived exosomes, leading to reduced PD-L1 expression and enhanced T-cell–mediated immune responses in gastric cancer (29). In addition, in lung adenocarcinoma, circulating exosomal miR-16-5p levels have been described as inversely correlated with PD-L1 expression and tumor burden, while *in vitro* restoration of miR-16-5p has been associated with reduced proliferation and migration and increased apoptosis (143).

However, it is important to emphasize that these findings derive from non-TGCT malignancies, and their relevance in TGCT remains to be established.

#### 6.3.2 CAR-T therapy: “hardwiring” cytotoxicity and overcoming suppressive tumor defenses

Chimeric antigen receptor T-cell (CAR-T) therapy is a form of adoptive cell therapy in which a patient’s T cells are collected, genetically engineered to express a synthetic receptor that recognizes tumor-associated antigens, expanded ex vivo, and reinfused to mediate antigen-specific cytotoxicity independently of classical MHC presentation.

In recent years, preclinical studies have explored the incorporation of microRNAs into CAR-T cell platforms as a strategy to modulate T-cell function. These approaches suggest that miRNA engineering may influence effector programs, including cytokine production and cytotoxic activity, thereby potentially enhancing anti-tumor responses.

For example, the integration of miR-155 into anti-CD19 CAR-T cells has been reported to improve anti-tumor activity in lymphoma models, in part through increased IFN-γ production (144). Similarly, miR-153 has been shown in colorectal cancer models to enhance CAR-T–mediated cytotoxicity by downregulating the immunoregulatory enzyme IDO1 (indoleamine 2,3-dioxygenase), a key mediator of immune suppression (145).

While these findings support the concept that miRNA modulation may augment CAR-T cell function, it is important to note that current evidence is largely limited to preclinical models in non-TGCT malignancies. Further studies are required to determine whether such strategies are applicable and effective in TGCT.

#### 6.3.3 miRNA mimics: restoring tumor-suppressor miRNAs to boost immunogenicity

miRNA mimics are synthetic, double-stranded RNA molecules designed to restore or boost the activity of a specific miRNA that is missing or downregulated in a cell, often a tumor-suppressive miRNA in cancer. By recapitulating endogenous miRNA function, they can repress target mRNAs and modulate multiple downstream pathways. These molecules can be delivered using a range of platforms, including lipid nanoparticles, polymers, viral vectors, and extracellular vesicles/exosomes.

In the context of cancer immunotherapy, miRNA mimics have been explored in preclinical models as a strategy to modulate immune-related pathways. These approaches have been associated with effects such as reduced expression of immune checkpoint molecules, modulation of suppressive myeloid programs, and alterations in cytokine signaling profiles that may favor anti-tumor immune responses.

Emerging evidence also suggests a potential role for miRNAs in the regulation of immune-related adverse events (irAEs). For example, lower levels of circulating or exosomal miR-146a have been associated with an increased risk of severe irAEs in patients receiving immune checkpoint inhibitors. In preclinical models, administration of miR-146a mimics has been reported to attenuate inflammatory responses and mitigate irAE-like toxicities (146).

From a clinical perspective, early-phase trials have evaluated miRNA mimics in oncology. Notably, the miR-16 mimic (MesomiR-1/TargomiRs), delivered via targeted nanoparticles, has been tested in patients with malignant pleural mesothelioma and non-small cell lung cancer, demonstrating feasibility and preliminary signals of activity, particularly in the latter setting.

However, it is important to emphasize that most of these findings derive from preclinical or early-phase clinical studies in non-TGCT malignancies, and their applicability in TGCT remains to be determined.

#### 6.3.4 miRNA inhibitors (antagomiRs): blocking oncomiRs that drive immune escape

Antagomirs are chemically modified, single-stranded antisense oligonucleotides designed to bind a specific miRNA and block its function. By targeting miRNAs implicated in tumor progression and immune regulation, these agents may modulate pathways associated with immune evasion, including myeloid cell expansion, macrophage polarization, immune checkpoint expression, and T-cell dysfunction.

In preclinical and early clinical settings, inhibition of selected oncomiRs has been associated with changes in the tumor immune microenvironment that could potentially enhance responsiveness to immunotherapy. However, these effects remain context-dependent and are not yet fully established across tumor types.

A representative example is cobomarsen (MRG106), an anti–miR-155 agent that has been evaluated in early-phase clinical studies in hematologic malignancies, including cutaneous T-cell lymphoma, chronic lymphocytic leukemia, diffuse large B-cell lymphoma, and adult T-cell leukemia/lymphoma (147, 148). These studies have demonstrated an acceptable safety profile and preliminary signals of clinical activity, particularly in T-cell lymphomas (147).

As with other miRNA-based therapeutic strategies, most available data derive from non-TGCT malignancies, and further investigation is required to determine their potential role in TGCT.

#### 6.3.5 Cancer vaccines: miRNAs as immune-potentiating players and response biomarkers

Cancer vaccines are immunotherapeutic strategies designed to enhance or prime anti-tumor immune responses, particularly by promoting antigen-specific T-cell activation. These approaches typically rely on the delivery of tumor-associated antigens or antigen-encoding platforms to antigen-presenting cells, especially dendritic cells (DCs), which subsequently initiate adaptive immune responses. Current vaccine platforms include peptide/protein-based vaccines, nucleic acid (DNA/RNA) vaccines, and cell-based approaches such as dendritic cell vaccines.

In this context, microRNAs have been investigated as potential biomarkers of immune activation and treatment response following vaccine-based therapies. In several malignancies, circulating miRNA profiles have been associated with clinical outcomes and immune activity. For example, in gastric cancer, elevated plasma miR-222 levels have been correlated with advanced disease stage and poorer survival, and may provide complementary information when combined with T-cell functional markers such as IL-2, TNFα, and IFNγ (36).

Similarly, in ovarian cancer, changes in circulating miRNAs—including miR-1228-5p, miR-193a-5p, and miR-375-3p—have been reported following administration of a glypican-3 (GPC3) peptide vaccine, with associations observed between these miRNA patterns and clinical response (149). In colorectal cancer, higher plasma levels of miR-6826 and miR-6875 have been associated with lower efficacy of HLA-A*2402 peptide vaccines (150).

Beyond their potential biomarker role, preclinical studies suggest that miRNAs may also influence vaccine-induced immune responses. For instance, overexpression of miR-155 in dendritic cells has been associated with enhanced immune activation and improved anti-tumor responses in murine models of breast cancer (25, 151).

Together, these findings suggest that miRNAs may serve both as indicators of vaccine-induced immune activity and as modulators of antigen presentation and T-cell priming. However, most of the available evidence derives from non-TGCT malignancies and preclinical models, and further studies are required to determine their relevance in TGCT.

## 7 miRNAS involved in testicular cancer and emerging strategies to enhance immunotherapy responsiveness

Reasoning on the diagnostic/prognostic miRNA landscape of testicular germ cell tumors and the current state of immunotherapy in TGCT, where checkpoint blockade has shown limited benefit in unselected patients, there is a clear need to identify actionable regulatory nodes capable of reshaping tumor–immune interactions. In this context, miRNAs offer an attractive bridge between TGCT biology and immunotherapy because they can simultaneously tune tumor-intrinsic programs (e.g., proliferation, stemness, DNA-damage response) and tumor-extrinsic immune pathways (e.g., antigen presentation, checkpoint expression, immune-cell exhaustion, and myeloid polarization).

In the final section of this review, we focus on selected microRNAs reported to be deregulated in TGCT and that have been implicated in the modulation of immunotherapy-related pathways (**Figure 1**), including immune checkpoints, the tumor microenvironment (TME), extracellular vesicles (EVs)/exosomes, and advanced platforms such as CAR-T. Where direct evidence in TGCT is limited, relevant findings from other malignancies are discussed as hypothesis-generating frameworks. We highlight overlapping candidates such as miR-34a, miR-17-5p, miR-378a-3p, the miR-301 family, and miR-375, discussing how each may influence immune escape or sensitivity and how they could be leveraged as biomarkers or therapeutic adjuvants. We then outline emerging translational strategies. including miRNA mimics to restore tumor-suppressive or immune-sensitizing miRNAs, antagomirs/inhibitors to hinder immunosuppressive circuits, EV-guided delivery for tissue-selective modulation of the TME, and engineered cell therapies (e.g., miRNA-augmented CAR-T products), conceptual strategies that may, in principle, help convert immunologically “cold” TGCT states, although this remains hypothetical in the absence of TGCT-specific validation. Despite transformative results of immune checkpoint inhibitors in multiple solid tumors, their impact in testicular germ cell tumors has been disappointingly limited in most clinical experiences to date, particularly in unselected relapsed/refractory disease. This apparent “failure” is biologically plausible: TGCTs frequently display features consistent with weak baseline immunogenicity and/or ineffective antigen presentation, while residual or treatment-shaped disease may be dominated by tumor-intrinsic survival programs and an immune microenvironment that is not optimally poised for productive T-cell priming and infiltration. In this setting, simply blocking PD-1/PD-L1 or related checkpoints may be insufficient because the principal barriers may lie upstream (insufficient immune recognition) and downstream (immunosuppressive tumor–immune crosstalk and adaptive resistance). A coherent hypothesis emerging from the overlap between TGCT-deregulated miRNAs and miRNAs known to modulate immunotherapy response, in other malignacies, is that miRNA biology may represent a potential regulatory layer that could be explored in future TGCT-specific studies to “reprogram” these bottlenecks, either by lowering tumor-intrinsic immune evasion, reshaping the tumor microenvironment (TME), and/or enabling response-adaptive clinical decision-making through liquid biopsy markers.

To facilitate interpretation and highlight the current evidence gaps, key miRNAs discussed in this review are summarized in **Table 1**, including their reported role in TGCT, immune-related mechanisms, level of evidence, and potential translational relevance.

**Table 1:** Candidate immunoregulatory miRNAs in TGCT.

miRNA	TGCT-associated role	Immune-related mechanism	Evidence level	Translational relevance
miR-34a	Tumor suppressor miRNA; not well studied in TGCT	In other malignancies, directly regulates PD-L1 expression and enhances T-cell–mediated anti-tumor responses	Indirect evidence (non-TGCT)	Potential candidate for checkpoint modulation; requires functional validation in TGCT
miR-17-5p	Member of the miR-17–92 cluster; implicated in proliferation and oncogenic signaling; limited TGCT-specific data	In other cancers, associated with immune evasion, modulation of T-cell responses, and cytokine signaling	Indirect evidence (non-TGCT)	Hypothesis-generating; potential role in tumor–immune interactions
miR-378a-3p	Poorly characterized in TGCT	In other malignancies, linked to macrophage polarization, angiogenesis, and immune suppression within the TME	Indirect evidence (non-TGCT)	Potential modulator of tumor-associated macrophages; requires validation in TGCT
miR-301 family	Not well defined in TGCT	Promotes immune evasion in other cancers through macrophage polarization, NF-κB activation, and cytokine signaling pathways | Indirect evidence (non-TGCT)		Conceptual candidate for immune escape mechanisms; currently hypothetical in TGCT
miR-375	Reported in TGCT; role remains unclear and potentially context-dependent	In other cancers, linked to immune signaling pathways and tumor–immune interactions	Biomarker-level evidence in TGCT; indirect immune evidence	Hypothesis-generating; potential biomarker and immunomodulatory role requiring further investigation

Immunoregulatory miRNAs shared between other cancers and TGCT, suggesting potential relevance in tumor–immune interactions.

### 7.1 MiR-34a

miR-34a is a biologically plausible therapeutic node in testicular germ cell tumors (TGCTs) because it sits at the intersection of tumor suppression, DNA-damage signaling, and immune evasion. In testicular cancer models, miR-34a and the related miR-449 family reduce proliferation and promote apoptosis, at least partly through p53-linked and E2F/pRB-associated circuitry, with repression of CDK6 and SIRT1 among the described effects; this fits the broader concept of miR-34 as a p53-responsive tumor-suppressive miRNA relevant to germ-cell homeostasis and stress responses (152, 153). In parallel, work from other malignancies showed that miR-34a can directly repress PD-L1/CD274 and participate in the p53→miR-34→PD-L1 axis, providing a mechanistic bridge between miRNA restoration and checkpoint biology (85, 112, 154–156). On this basis, miR-34a restoration could be proposed as a “checkpoint-sensitizing” intervention through at least four non-exclusive mechanisms: (a) direct PD-L1 repression on tumor cells (112, 157), (b) attenuation of suppressive PD-L1-positive myeloid states in the microenvironment (158, 159), (c) broader tumor-suppressor effects that may increase immune visibility by promoting stress and apoptosis (157, 160), and (d) rational combination with immune-priming modalities such as radiotherapy before or together with ICI (157). At present, however, there is no convincing evidence of a dedicated *in vivo* or clinical miR-34a restoration study in TGCT patients, and the TGCT-specific experimental evidence appears limited mainly to *in vitro* observations showing that enforced miR-34a/miR-449 activity suppresses proliferation and promotes apoptosis in testicular cancer cells (152, 153). Thus, proposing miR-34a restoration in TGCT is feasible, but still translational and inferential rather than validated in disease-specific animal models or patients. The most logical clinical niche would be relapsed/refractory seminoma or seminoma-predominant mixed TGCT, especially where PD-L1 dependence or an inflamed/exhausted T-cell context can be documented, and where this miRNA is commonly downregulated.

A major caution is that lowering PD-L1 alone is unlikely to be sufficient in TGCT. The pembrolizumab cohort explicitly linked weak clinical activity, at least in part, to low tumor mutational burden and limited baseline immunogenicity, and the nivolumab study likewise suggested that durable benefit may be enriched in tumors with higher TMB rather than in unselected cases (8, 10, 68, 161). Therefore, if miR-34a restoration is pursued, it should probably be embedded in combination strategies designed to “heat up” TGCT rather than used as monotherapy. Viable combinations include (1): radiotherapy-based immune priming plus miR-34a plus PD-1/PD-L1 blockade, because radiotherapy can promote immunogenic cell death and T-cell recruitment (162) (2). Epigenetic priming with hypomethylating agents (HMAs), such as decitabine or guadecitabine, which TGCT cells are unusually sensitive to, followed by miR-34a and immunotherapy, particularly in cisplatin-resistant embryonal carcinoma–containing disease. The second option is attractive because low-dose HMAs already show TGCT-specific preclinical activity in cells and xenografts and can reverse cisplatin resistance, making them one of the more disease-grounded priming platforms available in this tumor type (163, 164).

Safety is the other central issue. The clinical experience with MRX34 established proof-of-concept for miR-34 replacement in humans, but also showed that systemic liposomal delivery can trigger severe immune-mediated toxicity, including fatal events, despite dexamethasone premedication and schedule modification (85, 86). For that reason, any future TGCT-oriented miR-34 strategy should avoid simple recapitulation of MRX34. Safer translational options would include tumor-targeted or ligand-conjugated delivery systems, fully or heavily chemically modified miR-34a constructs with improved stability and reduced innate immune activation, dose step-up schedules, and biomarker-guided patient selection. These directions are supported by the broader RNA therapeutics literature, by nucleoside-modification studies showing reduced innate sensing, and by newer fully modified miR-34a platforms that achieved durable target repression and antitumor efficacy *in vivo* in non-TGCT models (165–167).

In summary, miR-34a restoration is a credible but still unproven therapeutic concept in TGCT. Principal advantages are a strong tumor-suppressor basis, a direct mechanistic connection to PD-L1 biology, and the possibility of simultaneously acting on tumor cells and the immune microenvironment. Main limitations are the absence of TGCT-specific therapeutic trials, the generally “cold” biology and low TMB refractory TGCT, and the toxicity lessons from MRX34. Overall, the best near-term use case would be a carefully selected seminoma or seminoma-predominant refractory TGCT, treated with safe, tumor-directed miR-34a delivery as part of a priming-plus-combination strategy, rather than systemic miR-34a replacement alone.

### 7.2 MiR-17-5p

miR-17-5p is a member of the miR-17–92 oncomiR cluster, broadly implicated in proliferation, survival and stem-like programs across different types of cancer (168, 169). In TGCT, miR-17–92 is most consistently linked to the embryonal carcinoma (EC)/pluripotent program and i’s downregulated upon differentiation (toward teratoma-like states), aligned with the loss of pluripotency factors such as OCT3/4 (OCT4) in mature teratoma (92, 170, 171).

From an immunotherapy perspective, as mentioned before, miR-17-5p can support immune evasion through PD-L1 upregulation (via indirect control of PD-L1 turnover). In colorectal cancer, tumor stem cell–derived exosomal miR-17-5p was reported to suppress anti-tumor immunity by targeting SPOP, thereby increasing PD-L1 and impairing immune control *in vivo* (172). While this is not TGCT-specific, it provides a tempting and plausible “immune axis” by which miR-17-5p inhibition could reduce PD-L1–linked immune escape in tumors where that pathway is active. In fact, strategies of anti-miR17-5p have been reported previously. The antagomir-17-5p inhibited growth *in vitro* and *in vivo* in therapy-resistant MYCN-amplified neuroblastoma, mechanistically linked to up-modulation of p21 and BIM, producing cell-cycle blockade and apoptosis (105). In addition, in the human clinical experience, it has been reported recently that the anti-miR-17 RGLS4326t is well tolerated in phase 1b with no serious adverse events in a small ADPKD (autosomal dominant polycystic kidney disease) cohort (173, 174). In addition, a next-generation anti-miR-17 (RGLS8429, “farabursen”) emerged after chemistry optimization to reduce off-target liabilities (reported mechanistically), and early-phase information/abstracts describe favorable tolerability in studied cohorts (175, 176). Therefore, these both evidences represent strong preclinical anti-tumor precedent for antagomir-17-5p in oncology, as well as real human tolerability experience for anti-miR-17-family chemistry. In TGCT, the most biologically plausible target for a possible antagomir-17-5p is the non-seminomatous, especially the embryonal carcinoma-dominant tumors or EC-like cell states, because miR-17-5p tracks with pluripotency - EC programs and is downregulated with differentiation. Accordingly, the less plausible target is the mature teratoma, because it is differentiated and typically lacks the pluripotency program and is managed primarily surgically. In fact, miR-17-5p expression is downregulated in teratomas (92, 170).

A key caveat for TGCT is that miR-17-5p is intertwined with cisplatin sensitivity in EC models. In the cisplatin-sensitive TERA human embryonal carcinoma cell lines, suppression of OCT4 (and the downstream miR-17/106b seed family milieu) is associated with increased cytoplasmic p21, which ultimately leads to a cisplatin chemoresistance phenotype (177, 178). As a consequence, indiscriminate anti-miR-17-5p use could inadvertently stabilize a p21-high, apoptosis-resistant state and potentially antagonizing cisplatin. In this regard, more defensible targets would be: 1) cisplatin-refractory or resistant NSGCT with EC-like features, where the clinical need is greatest and where immunotherapy sensitization would make more sense, and/or 2) those tumors showing evidence of immune-evasion circuitry potentially linked to miR-17-5p (e.g., PD-L1-high phenotypes where a miR-17-5p→SPOP→PD-L1-like mechanism is plausible), coupled with careful monitoring of p21 signaling states and response to cisplatin. Conversely, in cisplatin-sensitive EC-rich disease, an anti-miR-17-5p strategy could be risky unless there is a compelling immune rationale and strong preclinical synergy data demonstrating no loss of platinum efficacy.

#### 7.2.1 Priming approaches to boost immunotherapy

In addition, since TGCT immune responsiveness is generally complex and depends on the microenvironment immunogenicity, miR-17-5p inhibition is more plausible as a combination strategy than as monotherapy and since TGCT is considered as a “cold” or poorly inflamed tumor, “immune priming” approaches can supply the missing ingredients for effective checkpoint blockade, namely antigen release, type-I interferon signaling, dendritic-cell activation, and T-cell recruitment, thereby reducing the risk that PD-1/PD-L1 targeting (or PD-L1 downregulation via anti-miR approaches) is biologically insufficient. As examples, clinically and preclinically, radiotherapy is one of the best-supported priming modalities because it can increase tumor antigen availability and inflammatory signaling and has shown mechanistic and translational synergy with immune checkpoint inhibitors (179). Additionally, complementary priming can be achieved with immunogenic cell death (ICD)–competent chemotherapy, exemplified by oxaliplatin inducing key ICD hallmarks (e.g., calreticulin exposure) more robustly than cisplatin in benchmark models (180). Another example is the direct activation of innate sensing within tumors that has also been clinically explored via intratumoral TLR9 (Toll-like 9 receptor) agonism (e.g., SD-101) combined with pembrolizumab in melanoma, supporting the concept of local IFN-driven priming to sensitize to PD-1 blockade (181). Even when TLR9 agonism has not been tried in TGCT, there is evidence of TLR9 expression in normal and specially in cancer testis tissue, which is a rationale for testing this strategy in TGCT (182). Similarly, intratumoral STING agonism (Stimulator of interferon genes) has been tested in combination with anti-PD-1 (spartalizumab) in a phase 1b study in solid tumors, further reinforcing STING-IFN axis activation as a priming route (183). In this last work TGCT is not specifically mentioned, but they included several types of solid tumors, which leaves an open door to include other solid tumors such as TGCTs. Finally, DNA-damage–linked priming via PARP inhibition plus radiotherapy can activate the cGAS-STING pathway and increase T-cell chemoattractants (e.g., CCL5/CXCL10), enhancing anti-PD-L1 activity, an example of multi-hit priming plus checkpoint blockade (184). This approach has been tried in small cell lung cancer, both *in vitro* and *in vivo*. Nonetheless, although PARP inhibitors have not shown meaningful clinical activity in testicular cancer, specifically in platinum-refractory TGCT (185), a better molecular selection of patients could be done to prime immune response and combine inhibition of miR-17-5p and anti-PD-L1 strategy.

### 7.3 miR-378a-3p

miRNA profiling of cisplatin-sensitive versus cisplatin-resistant TGCT cell models has identified miR-378a-3p among the miRNAs downregulated in resistant non-seminomatous contexts, and this low-expression state was proposed as part of a biomarker panel associated with chemoresistance, a more aggressive phenotype, and potentially metastatic dissemination, especially in non-seminomatous TGCTs (NSGCTs). For this reason, reduced miR-378a-3p expression is currently best linked to treatment failure (186). Evidence from other tumor types strengthens the view of miR-378a-3p as a context-dependent tumor suppressor that can influence both therapy response and immune escape. In hepatocellular carcinoma, restoration of miR-378a-3p inhibited proliferation and migration, increased apoptosis, and reduced PD-L1 and STAT3 expression, which connects this miRNA to an immune-checkpoint-relevant axis. Similarly, in ovarian cancer, miR-378a-3p restoration increased cisplatin sensitivity through repression of MAPK1 and GRB2, while in colorectal cancer models this miRNA reduced tumor growth, clonogenicity, stem-like features, and malignant progression. Collectively, these data suggest that miR-378a-3p restoration may exert a dual effect: re-sensitizing tumor cells to cytotoxic stress and attenuating immune evasion pathways, which is highly relevant to TGCT (154, 187, 188). Translating these findings to TGCT, one may hypothesize that restoring miR-378a-3p in cisplatin-resistant NSGCT could simultaneously weaken survival signaling and reduce checkpoint-mediated immune suppression, particularly if PD-L1/STAT3 regulation is conserved in this disease. However, this remains a biologically plausible extrapolation rather than a demonstrated TGCT mechanism, because no published *in vitro*, *in vivo*, or clinical study has yet directly tested therapeutic restoration of miR-378a-3p in TGCT models or patients. Therefore, its current role in TGCT is best framed as a promising translational hypothesis supported by biomarker data in TGCT and functional evidence from other cancers.

#### 7.3.1 How miR-378a-3p restoration could support TGCT treatment, particularly immunotherapy

Given that clinical studies of pembrolizumab and nivolumab in heavily pretreated germ cell tumors have shown minimal response rates overall, possibly because TGCT generally have low tumor mutational burden (TMB) and limited neoantigenicity, miR-378a-3p restoration alone would probably be insufficient, even if it lowers PD-L1, because TGCT immune resistance is not driven by a single checkpoint pathway. A more rational concept is a combination strategy in which miR-378a-3p acts as a tumor-intrinsic sensitizer while another intervention increases tumor immunogenicity or T-cell recruitment. The strongest current partner in TGCT is probably epigenetic priming with DNA methyltransferase inhibition, especially guadecitabine, which has shown preclinical and early clinical relevance in platinum-refractory germ cell tumors. In embryonal carcinoma and refractory TGCT models, guadecitabine not only restored cisplatin sensitivity but also induced immune-related pathways, HLA class I expression, interferon signaling, and cancer-testis antigens, providing exactly the type of “tumor heating” that could complement a PD-L1-lowering miRNA (66, 164, 189). Accordingly, a plausible therapeutic sequence for cisplatin-resistant NSGCT, especially embryonal carcinoma-rich or yolk sac tumor-containing disease, would be: (i) epigenetic priming with guadecitabine, (ii) restoration of miR-378a-3p to suppress PD-L1/STAT3-like immune escape and possibly re-sensitize to platinum, and (iii) PD-1/PD-L1 blockade. In selected molecularly enriched cases, further combinations such as PARP inhibition plus checkpoint blockade may also be reasonable, particularly in tumors with high TMB or DNA-repair-related alterations, although this remains highly exploratory in TGCT.

Regarding delivery strategies, systemic, non-targeted miRNA administration raises concerns about degradation, off-target distribution, and immune toxicity. Therefore, tumor-directed delivery is essential. Among the available platforms, engineered exosomes/extracellular vesicles (EVs) are especially attractive for miR-378a-3p or miR-378a-3p mimics delivery because they naturally protect miRNA cargo, can be modified for tumor tropism, and are increasingly viewed as promising carriers for cancer-directed nucleic acid therapy. Nonetheless, their limitations are also important: heterogeneity, manufacturing complexity, loading efficiency, and biodistribution control remain unresolved translational hurdles (190–192). More conventional delivery systems also deserve mention. Nanoparticles, particularly lipid nanoparticles, remain the most mature platform for active miRNA delivery because they can be functionalized with tumor-targeting ligands and have a clearer manufacturing path than exosomes. In TGCT, a CLDN6-targeted nanoparticle carrying miR-378a-3p mimic would be a particularly logical design for disseminated disease (193). Another interesting platform to deliver miR-378a-3p could be hydrogels, which are soft, water-swollen three-dimensional polymer networks that can be engineered to be injectable, biodegradable, and responsive to local stimuli such as pH, enzymes, or temperature. In oncology, their main value is to act as localized depots that retain therapeutics at the tumor site and enable sustained, spatially controlled release, thereby reducing systemic exposure. For RNA therapeutics, this is especially useful because hydrogels can protect miRNAs, siRNAs, mRNA, or aptamers from rapid degradation, improve local retention, and facilitate combination delivery with other agents such as chemotherapy (to make TGCT “hot”) or immunotherapy. Preclinical studies have reported successful hydrogel-based RNA delivery in several tumors, including triple-negative breast cancer, where RNA-triple-helix hydrogels or miRNA/siRNA-loaded systems produced marked tumor regression, as well as melanoma and breast cancer xenografts using localized siRNA-releasing hydrogels. Overall, hydrogels are best viewed as promising locoregional RNA carriers for accessible tumors, local or residual lesions. In contrast, they are not ideal platforms for widely disseminated metastatic disease (194–198).

These strategies are unlikely to be relevant for the majority of newly diagnosed, curable TGCTs. Their main niche would be relapsed or platinum-refractory metastatic disease, where standard therapy has failed and new biology-driven options are needed. Among TGCT subtypes, the most rational setting is non-seminomatous TGCT, especially cisplatin-resistant NSGCT enriched for embryonal carcinoma and/or yolk sac tumor features, because this is the context in which miR-378a-3p downregulation has been linked to aggressive disease and where CLDN6-targeted approaches are especially attractive (186, 199, 200). Overall, restoration of miR-378a-3p is a biologically coherent strategy in TGCT. The main pros are its potential to couple tumor-intrinsic re-sensitization to cisplatin with reduced immune escape, especially through PD-L1/STAT3-like pathways suggested by non-TGCT models. The main cons are equally important: in TGCT, therapeutic restoration of miR-378a-3p has not yet been directly validated, anti-PD-1 monotherapy is already weak in most unselected cases, and successful translation will likely require combination therapy plus targeted delivery. Thus, miR-378a-3p should currently be viewed as a promising adjunctive immuno-sensitizing candidate for refractory NSGCT, particularly in combination with epigenetic priming, checkpoint blockade, and CLDN6-oriented delivery or cell therapy, rather than as a standalone immunotherapy solution.

### 7.4 miR-301a

In testicular germ cell tumors (TGCTs), miR-301/miR-301a has been linked more to tumor differentiation state than to the canonical embryonic/pluripotency program. Early microRNA profiling studies showed that hsa-miR-301 is enriched in more differentiated histologies, including teratoma, yolk sac tumor, and spermatocytic tumor (formerly spermatocytic seminoma), whereas it is comparatively absent from embryonal carcinoma, a pluripotent non-seminomatous component. Thus, within TGCT biology, miR-301a-3p appears to align with a more differentiated phenotype rather than with the stem-like malignant program that characterizes embryonal carcinoma. This makes miR-301a-3p potentially interesting not as a pan-TGCT biomarker, but as a lineage- and context-dependent mediator of the differentiated/non-pluripotent compartment (92, 170, 201), (**Figure 1**).

Outside TGCT, however, miR-301a-3p is better established as an oncomiR with immunoregulatory functions. In pancreatic cancer, hypoxic tumor-derived exosomal miR-301a-3p promotes M2 macrophage polarization through PTEN suppression and PI3Kγ/AKT pathway activation, thereby fostering invasion, metastasis, and immune suppression. In glioblastoma, hypoxic exosomal miR-301a activates Wnt/β-catenin signaling by targeting TCEAL7, promoting radioresistance. Additional studies in other tumor types support a broader oncogenic role for miR-301a through PTEN/AKT signaling, and suppression of miR-301a activity has reduced proliferation or tumor growth in preclinical models. Collectively, these data provide a plausible mechanistic framework by which miR-301a-3p could also contribute to immune escape in TGCT, even though direct TGCT-specific proof is still lacking (202–205). There is no evidence at the moment that miR-301a-3p might be secreted via tumor exosomes by TGCT cells. However, we do know that they release miRNA-containing extracellular vesicles (EVs), and these EVs can be internalized by fibroblasts, endothelial cells, and macrophage-like cells, inducing phenotypic and transcriptomic changes in the tumor microenvironment. This has been demonstrated for the canonical TGCT-associated miRNA clusters (miR-371~373 and miR-302/367), but for miR-301a it remains to be proven (206). This distinction matters because the TGCT microenvironment is not immunologically uniform. Seminoma generally shows a more inflamed milieu, with higher immune-cell infiltration, whereas non-seminomatous tumors are typically less inflamed and more heterogeneous. In TGCT, macrophage biology is already known to influence tumor behavior: a miR-125b/CSF1-CX3CL1 axis has been shown to regulate tumor-associated macrophage recruitment and TGCT growth, demonstrating that miRNA-mediated tumor–stroma crosstalk is biologically relevant in this disease. Thus, if miR-301a-3p were shown to be active in differentiated TGCT components, one reasonable hypothesis is that it could reinforce an immunosuppressive macrophage-rich niche, particularly in differentiated non-seminomatous areas (207, 208).

Inhibiting miR-301a-3p itself or intercepting its PTEN/PI3Kγ/AKT downstream axis can remodel the tumor microenvironment (TME) in a way that should, in principle, improve responsiveness to immunotherapy. The study in pancreatic cancer (202) is important because it directly links miR-301a to a macrophage-centered immunosuppressive circuit rather than only to tumor-cell intrinsic growth. Similar exosome-to-macrophage effects were later shown in esophageal squamous cell carcinoma, again through PTEN inhibition and PI3K/AKT activation, with enhanced angiogenesis and a pro-tumor macrophage phenotype (209). From a translational standpoint, the best indirect evidence that the axis miR-301a-3p–macrophage behavior can be exploited to improve immunotherapy comes from targeting the downstream pathway, especially PI3Kγ in myeloid cells. In mouse tumor models, pharmacologic inhibition of PI3Kγ reprogrammed suppressive macrophages, increased T-cell recruitment/function, and restored sensitivity to checkpoint blockade. A landmark Nature study showed that selective PI3Kγ inhibition reshaped the TME and overcame resistance to checkpoint blockade. A companion study established PI3Kγ as a molecular switch controlling macrophage-mediated immune suppression. In humans, early-phase clinical development of the selective PI3Kγ inhibitor *Eganelisib* (IPI-549) has provided proof that this macrophage-directed strategy is clinically feasible, and combination studies with checkpoint inhibitors have been pursued in solid tumors such as metastatic triple-negative breast cancer. Thus, while this is not an anti-miR-301a trial, it is a strong mechanistic support for targeting the same immunosuppressive node that miR-301a-3p is known to regulate in other tumors (210–213). Regarding TGCT, from a mechanistical point of view, one could envision three escalating levels of intervention. First, direct anti-miR-301a-3p inhibition using a chemically stabilized antagomir, LNA-anti-miR, or nanoparticle/lipid-delivered antisense oligonucleotide directed at miR-301a-3p in tumor cells and/or tumor-derived EVs. Second, pathway interception downstream, particularly PI3Kγ inhibition in macrophages or possibly broader PI3K/AKT pathway modulation when justified by tumor biology. Third, addition of an immune-priming partner, for example chemotherapy, radiotherapy, or another microenvironment-modulating agent, to increase antigen release, danger signaling, and T-cell recruitment before or during PD-1/PD-L1 blockade. This layered approach is more biologically plausible than expecting anti-miR-301a alone to convert an immune-poor TGCT into a fully “hot” tumor. The general principle is supported by evidence that PTEN loss is associated with a non-T-cell-inflamed phenotype and inferior response to anti-PD-1 therapy, whereas reversing PI3K pathway-driven immune suppression can improve antitumor immunity (214–216). Since PD-L1 expression is not the highest in the differentiated subtypes of TGCT, one might consider that the intended benefit may lie more in macrophage/TME reprogramming than in simply lowering tumor-cell PD-L1. Additionally, potential toxicities and translational risks should also be acknowledged. Direct anti-miR therapy can produce off-target hybridization effects, innate immune activation, hepatic uptake, complement activation, infusion reactions, and class-specific toxicities related to oligonucleotide chemistry and delivery systems. On-target toxicity is also conceivable because miR-301a participates in inflammatory and tissue homeostasis networks beyond cancer. If the strategy instead uses PI3Kγ inhibitors, known early clinical toxicities have included fatigue, nausea, liver enzyme elevations, rash, and other adverse events typical of pathway inhibitors, while combination with checkpoint blockade may increase immune-related toxicity. In a young TGCT population, fertility, gonadal endocrine function, and long-term survivorship issues would deserve particular attention, even though no direct evidence currently indicates that anti-miR-301a-3p would uniquely damage the testis (212, 217).

In summary, there is no TGCT-specific study yet showing that inhibition of miR-301a-3p improves the TME and sensitizes TGCT to immunotherapy. Nevertheless, the idea is biologically plausible and experimentally justified because: (i) TGCTs do exchange miRNA cargo with the TME via EVs, (ii) TGCT growth can be modulated through a miRNA–macrophage axis, and (iii) in other cancers, miR-301a-3p and its PTEN/PI3Kγ/AKT axis clearly promote macrophage-mediated immune suppression. Therefore, an anti-miR-301a-3p strategy should be proposed as a hypothesis-generating, combination-based strategy, most relevant to differentiated non-seminomatous TGCT, with the expectation that TME reprogramming plus immune priming plus checkpoint blockade would be more effective than miR-301a inhibition alone.

### 7.5 miR-375

As we mentioned above, in TGCT miR-375 is best supported as a teratoma-enriched biomarker. Tissue studies consistently show strong miR-375 expression in teratoma, whereas circulating performance in serum/plasma is far less robust. *Belge* et al. found that serum miR-375-3p did not reliably distinguish teratoma from other GCT components or controls, arguing against its use as a single liquid-biopsy marker (218). By contrast, studies integrating miR-375 with miR-371a-3p have been more informative: *Nappi* et al. reported that combined plasma evaluation improved identification of teratoma and active malignant GCT components (219), and *Kremer* et al. showed in post-chemotherapy retroperitoneal lymph node dissection (RPLND) tissue that miR-371a-3p plus miR-375-5p could separate viable tumor and teratoma from necrosis/fibrosis with high accuracy (220). Recent reviews therefore position miR-375 as a useful “teratoma-complement” to miR-371 rather than a serum surrogate sufficient on its own (221).

Beyond its diagnostic utility in TGCTs, miR-375 has emerged as a crucial predictive biomarker of response to immunotherapy, cancer vaccines, and cellular therapies across various malignancies. Interestingly, the prognostic implication of miR-375 expression appears to be highly context-dependent. In hematological malignancies, increased levels of miR-375 have been correlated with favorable responses to anti-CD19 CAR-T cell therapy in patients with B-cell acute lymphoblastic leukemia (B-ALL), although the mechanistic basis remains incompletely defined and appears to come from transcriptomic/network analyses rather than direct causal validation (25). Similarly, in solid tumors such as ovarian clear cell carcinoma (OCCC), elevated serum levels of extracellular miR-375-3p has been identified as predictive biomarker of positive clinical responses to the Glypican-3 (GPC3) peptide vaccine. Importantly, functional annotation linked these serum miRNAs to interferon-related pathways, and the authors noted they were not simply tumor-tissue-derived markers (149). Conversely, in the context of immune checkpoint inhibitors (ICIs), low baseline serum levels of miR-375 in patients with advanced non-small cell lung cancer (NSCLC) have been significantly associated with a clinical benefit (partial or complete response) to the anti-PD-1 antibody Nivolumab. The investigators linked miR-375 to immune-relevant nodes such as JAK2, TGF-β2, Wnt, and Hippo/YAP1 pathways (111). This dichotomy suggests that miR-375 is not merely a passive byproduct of tumor shedding, but an active participant in shaping the tumor immune microenvironment. At present, the role of miR-375 in TGCT immune modulation remains largely speculative and should be interpreted as a hypothesis-generating observation. For TGCT, these associations are biologically intriguing but should not yet be overinterpreted. At present, there is no established vaccine, cell therapy, or ICI strategy specifically validated against teratoma. Thus, miR-375 cannot currently be viewed as a predictive biomarker of immunotherapy efficacy in TGCT; rather, its immediate value remains histologic stratification, especially for distinguishing residual teratoma from necrosis/fibrosis when combined with miR-371. Actually, immunotherapy for viable TGCT is being revisited through target-based approaches, particularly CLDN6-directed therapy. Preclinically, CLDN6 is expressed in several germ cell tumor subtypes, and a CLDN6 antibody-drug conjugate showed activity in GCT models, with stronger effects in seminoma/embryonal carcinoma than in yolk-sac tumor. Clinically, the phase 1/2 BNT211–01 study of CLDN6 CAR-T cells ± CARVac (an RNA vaccine that amplifies CAR-T expansion) reported encouraging activity, with the highest response rate in germ cell tumors among the enrolled solid tumors. This is attractive for viable non-teratomatous TGCT, especially embryonal carcinoma and CLDN6-positive disease. However, it is not a teratoma-specific strategy, and mature teratoma may be less suitable because it is biologically differentiated, often surgically resectable, and may not present the same surface-antigen/immunogenic landscape as active malignant GCT (200, 222). However, the high expression of miR-375, combined with specific germ-cell antigens, opens the door for alternative targeted immunotherapies. For instance, the GPC3 antigen, which has shown promise in ovarian cancer vaccines, is frequently expressed in yolk sac tumors and certain TGCT subtypes (223). A GPC3-directed peptide vaccine or CAR-T cell approach could theoretically be efficacious in TGCTs. In this scenario, high miR-375 expression in the teratoma component could serve as a predictive biomarker for vaccine/CAR-T efficacy, just as it does in OCCC and B-ALL, because the low PD-L1 environment maintained by miR-375 would allow the engineered or vaccine-primed T-cells to attack without being immediately deactivated by checkpoint engagement. Furthermore, the integration of miR-371a-3p into this framework could be therapeutically transformative. While miR-371 is an established diagnostic marker, the miR-371–373 cluster is also known to regulate oncogenic signaling and modulate the tumor immune microenvironment (TIME) in other cancers. Specifically, overexpression of miR-373 suppresses the expression of MICA (MHC class I polypeptide-related sequence A) on the surface of tumor cells. By reducing the presence of this ligand, the tumor drastically lowers its susceptibility to recognition and destruction by NK cells, consolidating an immune evasion phenotype in the microenvironment (17). Therefore, If a patient’s circulating profile reveals high miR-371a-3p (viable tumor) but low miR-375, the tumor may have uninhibited PD-L1 expression, suggesting a potential, albeit rare, rationale for ICI therapy (considering a combined regime as mentioned above several times, so that this tumor became “hot”). Conversely, a profile showing low miR-371a-3p and high miR-375 (pure teratoma) would confirm that traditional ICIs are futile, steering the clinical decision strictly toward pcRPLND or trials involving cellular therapies (e.g., CAR-T). Thus, today, the best use of the miR-371 + miR-375 combination is diagnostic/pathology-guiding, not therapeutic selection. A future therapeutic value might be still conceivable.

## 8 Integrating miRNA biology and tumor immunity in TGCT: implications for future immunotherapeutic strategies

This review sought to integrate three areas that are often considered separately—TGCT biology, the limited efficacy of immunotherapy in this disease, and the emerging role of microRNAs (miRNAs) in immune regulation—into a unified, TGCT-centered framework. The central premise is not that miRNA-based strategies are ready for clinical implementation in TGCT, but rather that miRNA-mediated regulation may help explain key features of the TGCT tumor immune microenvironment (TME) and provide a rationale for future, disease-specific investigation.

A recurring observation across the literature is that TGCTs occupy an intermediate position between immune-infiltrated and functionally immunosuppressed tumors. While certain subtypes, particularly seminomas, can display substantial immune cell infiltration, this does not consistently translate into effective anti-tumor immunity or responsiveness to immune checkpoint blockade. This apparent paradox is likely rooted in the unique immunobiology of the testis, where elements of immune privilege—such as restricted antigen presentation, tolerogenic antigen-presenting cells, and local immunosuppressive signaling—are only partially disrupted during tumorigenesis. As a result, TGCTs may represent a form of “immune-dysregulated” rather than purely “immune-cold” tumors, in which both immune activation and dominant inhibitory signals coexist.

Within this context, the limited clinical activity of immune checkpoint inhibitors in TGCT can be understood as the consequence of multiple converging factors, including low tumor mutational burden, reduced neoantigen diversity, and a TME shaped by residual mechanisms of immune tolerance. These features distinguish TGCT from more immunogenic malignancies and suggest that strategies relying solely on checkpoint blockade may be insufficient to induce durable responses, particularly in the relapsed or cisplatin-refractory setting.

miRNAs provide a potential layer of regulation that could contribute to this complex immune landscape. In other cancer types, miRNAs have been shown to modulate key processes relevant to anti-tumor immunity, including checkpoint molecule expression, macrophage polarization, dendritic cell function, and cytokine signaling. However, as highlighted throughout this review, the majority of these data derive from non-TGCT models. In TGCT specifically, current evidence is largely limited to biomarker studies or descriptive expression analyses, with relatively few functional investigations directly linking miRNAs to immune modulation.

This distinction has important implications. While it is biologically plausible that miRNAs contribute to immune escape mechanisms in TGCT, their precise roles remain to be defined in disease-specific systems. Accordingly, miRNA-mediated pathways discussed here should be interpreted as hypothesis-generating rather than as established drivers of TGCT immunobiology. Bridging this gap will require well-designed functional studies using TGCT cell lines, organoid systems, and patient-derived samples, ideally incorporating immune co-culture models to capture interactions within the TME.

An additional layer of complexity arises from the marked heterogeneity within TGCT. Seminomas, non-seminomatous germ cell tumors (NSGCTs), and mature teratomas differ not only in their histopathological features but also in their immune composition and potential responsiveness to immunomodulatory strategies. For example, mechanisms involving immune cell recruitment or checkpoint modulation may be more relevant in seminomas, whereas cisplatin-resistant NSGCTs may be characterized by more pronounced immunosuppressive signaling. Mature teratomas, in contrast, may remain largely refractory to immune-based approaches due to low proliferative activity and limited immunogenicity. Future studies should therefore consider TGCT subtype–specific contexts when evaluating miRNA function and therapeutic potential.

From a translational perspective, miRNAs may have the greatest near-term impact as biomarkers rather than as direct therapeutic agents. Circulating miRNAs such as miR-371a-3p already demonstrate high sensitivity and specificity for TGCT detection and monitoring. Whether similar approaches can be extended to capture immune-related states—such as immune activation, suppression, or therapy resistance—remains an open but promising question. In contrast, therapeutic strategies involving miRNA mimics, antagomirs, or targeted delivery systems (e.g., nanoparticles or exosome-based platforms) are conceptually attractive but remain at an early stage of development in the context of TGCT and should be regarded as long-term objectives.

Looking forward, several priorities emerge for the field. First, there is a need for systematic characterization of the TGCT immune microenvironment at single-cell resolution, integrating transcriptomic, epigenetic, and miRNA profiling. Second, functional validation of candidate miRNAs in TGCT-specific models is essential to move beyond correlative observations. Third, combination strategies that integrate miRNA modulation with established or emerging immunotherapies should be explored cautiously, with careful attention to biological plausibility and subtype specificity. Finally, the design of future clinical studies should incorporate biomarker-driven approaches to better identify patient subsets that may benefit from immune-based interventions.

In conclusion, TGCT represents a biologically distinct tumor type in which conventional paradigms of cancer immunotherapy do not fully apply. miRNAs offer a potentially informative lens through which to reinterpret the TGCT immune microenvironment, but their role remains incompletely defined. Rather than providing immediate therapeutic solutions, the integration of miRNA biology into TGCT research highlights critical gaps in our understanding and points toward a more nuanced, mechanism-driven approach to immunomodulation in this disease.

## 9 Conclusions

In conclusion, immunotherapy has not yet fulfilled its promise in testicular germ cell tumors, particularly in unselected patients with relapsed or platinum-refractory disease. Although these tumors frequently express immune checkpoints such as PD-L1 and may contain tumor-infiltrating lymphocytes, this apparent immunological activity has not yet translated into consistent clinical benefit with conventional immune checkpoint blockade. As synthesized throughout this review, several convergent factors help explain this discrepancy: the generally low tumor mutational burden and limited neoantigen repertoire of TGCTs; the immune-privileged nature of the testis and its inherited tolerance mechanisms; the complexity and heterogeneity of the tumor microenvironment; the imperfect predictive value of static biomarkers such as PD-L1 immunohistochemistry; and the fact that, in many cases, the major barriers to immune efficacy likely reside not only at the level of checkpoint signaling, but also upstream in insufficient immune priming and downstream in adaptive immune escape, suppressive myeloid crosstalk, and defective antigen presentation.

Within this framework, miRNAs emerge as particularly attractive tools because they occupy a mechanistic position above many of these resistance layers. Rather than acting on a single immune node, miRNAs can simultaneously influence tumor-cell survival, pluripotency, DNA-damage response, antigen presentation, cytokine signaling, checkpoint expression, macrophage polarization, dendritic-cell function, T-cell exhaustion, and extracellular-vesicle-mediated communication. This pleiotropic regulatory capacity is especially relevant in TGCT, where monotherapies directed at a single axis have shown limited efficacy. Accordingly, the greatest translational promise of miRNA-based strategies is unlikely to lie in their isolated use, but in their rational integration with other therapies capable of increasing tumor immunogenicity. In this regard, miRNA mimics, antagomirs, engineered extracellular vesicles, or miRNA-augmented cellular therapies could be combined with epigenetic priming, radiotherapy, immunogenic chemotherapy, STING/TLR agonism, vaccines, or CAR-T-based approaches to convert immunologically “cold,” immune-privileged, or poorly responsive TGCT states into tumors more permissive to durable anti-tumor immunity.

The path forward now requires a more precise and stepwise translational agenda. First, TGCT-specific functional studies are needed to validate the causal immunological roles of candidate miRNAs such as miR-34a, miR-17-5p, miR-378a-3p, miR-301a, and miR-375 in relevant seminomatous and non-seminomatous models. Second, future research should move beyond descriptive biomarker associations and establish mechanism-based therapeutic combinations capable of simultaneously enhancing antigenicity, relieving immune suppression, and preserving anti-tumor specificity. Third, delivery remains a decisive issue: safer and more tumor-directed platforms, such as ligand-targeted nanoparticles, engineered exosomes, or localized biomaterial-based systems, will be essential to reduce off-target effects and avoid the immune toxicities that limited early-generation miRNA therapeutics. Fourth, biomarker-driven patient selection must become central, integrating miRNA profiles with histology, molecular subtype, immune contexture, and features such as DNA-repair alterations, MSI, or exceptional mutational burden. Finally, early-phase clinical trials should prioritize biologically enriched subgroups and combination regimens rather than repeating the largely disappointing strategy of testing checkpoint inhibitors alone in unselected refractory populations.

If successfully developed, this miRNA-guided immunotherapeutic framework could offer more than a new salvage option; it could redefine how refractory TGCT is biologically stratified and treated. The potential benefit to the patient is especially meaningful in this disease because TGCT affects adolescents and young adults at a stage of life marked by education, professional development, fertility, sexuality, family planning, and long-term survivorship concerns. For these patients, treatment failure does not only threaten survival, but also disproportionately compromises quality of life over decades. Therefore, improving immunotherapy responsiveness through miRNA-based combination strategies is not merely a technical or mechanistic goal: it represents a potential long-term objective that will require careful biological validation and clinical investigation to reduce toxicity, extend survival, preserve function, and ultimately prevent young patients from losing health, life opportunities, and, too often, life itself at an early age.

## References

[1] Singla N Bagrodia A Baraban E Fankhauser CD Ged YMA . Testicular germ cell tumors. JAMA. (2025) 333:793–803. doi: 10.1001/jama.2024.27122 39899286

[2] Oldenburg J Berney DM Bokemeyer C Climent MA Daugaard G Gietema JA . Testicular seminoma and non-seminoma: ESMO-EURACAN clinical practice guideline for diagnosis, treatment and follow-up ☆. Ann Oncol. (2022) 33:362–75. doi: 10.1016/j.annonc.2022.01.002 35065204

[3] Abughanimeh O Teply BA . Current management of refractory germ cell tumors. Curr Oncol Rep. (2021) 23:101. doi: 10.1007/s11912-021-01093-z 34269906

[4] Mehr F Emtiazi N Zolfi E . Advances in cell therapy for testicular cancer: a comprehensive overview of immunotherapy and stem cell therapy. Tissue Cell. (2025) 98:103169. doi: 10.1016/j.tice.2025.103169 41067171

[5] Perrier A Didelot A Laurent-Puig P Blons H Garinet S . Epigenetic mechanisms of resistance to immune checkpoint inhibitors. Biomolecules. (2020) 10:1061. doi: 10.3390/biom10071061 32708698 PMC7407667

[6] Kumar S Sarthi P Mani I Ashraf M Kang M Kumar V . Epitranscriptomic approach: To improve the efficacy of ICB therapy by co-targeting intracellular checkpoint CISH. Cells. (2021) 10:2250. doi: 10.3390/cells10092250 34571899 PMC8466810

[7] García-Giménez J Saadi W Ortega A Lahoz A Suay G Carretero J . miRNAs related to immune checkpoint inhibitor response: A systematic review. Int J Mol Sci. (2024) 25:1737. doi: 10.3390/ijms25031737 38339019 PMC10855819

[8] Chovanec M Mardiak J Mego M . Immune mechanisms and possible immune therapy in testicular germ cell tumors. Andrology. (2019) 7:479–86. doi: 10.1111/andr.12656 31169364

[9] Kalavska K Schmidtova S Chovanec M Mego M . Immunotherapy in testicular germ cell tumors. Front Oncol. (2020) 10:573977. doi: 10.3389/fonc.2020.573977 33072608 PMC7542989

[10] Tsimberidou A Vo H Subbiah V Janku F Piha-Paul S Yilmaz B . Pembrolizumab in patients with advanced metastatic germ cell tumors. Oncol. (2021) 26:558–e1098. doi: 10.1002/onco.13682 33491277 PMC8265349

[11] Schepisi G Gianni C Cursano M Gallà V Menna C Casadei C . Immune checkpoint inhibitors and chimeric antigen receptor (CAR)-T cell therapy: Potential treatment options against testicular germ cell tumors. Front Immunol. (2023) 14:1118610. doi: 10.3389/fimmu.2023.1118610 36860862 PMC9968831

[12] Liu Q Lian Q Lv H Zhang X Zhou F . The diagnostic accuracy of miR-371a-3p for testicular germ cell tumors: A systematic review and meta-analysis. Mol Diagn Ther. (2021) 25:273–81. doi: 10.1007/s40291-021-00521-x 33886084

[13] Chavarriaga J Hamilton RJ . miRNAs for testicular germ cell tumors: Contemporary indications for diagnosis, surveillance and follow‐up. Andrology. (2023) 11:628–33. doi: 10.1111/andr.13337 36373757

[14] Leão R Albersen M Looijenga LHJ Tandstad T Kollmannsberger C Murray MJ . Circulating microRNAs, the next-generation serum biomarkers in testicular germ cell tumors: A systematic review. Eur Urol. (2021) 80:456–66. doi: 10.1016/j.eururo.2021.06.006 34175151

[15] Fankhauser CD Nuño MM Murray MJ Frazier L Bagrodia A . Circulating microRNAs for detection of germ cell tumors: A narrative review. Eur Urol Focus. (2022) 8:660–2. doi: 10.1016/j.euf.2022.04.008 35537936

[16] Zare E Yaghoubi SM Khoshnazar M Dargahlou SJ Machhar JS Zheng Z . MicroRNAs in cancer immunology: Master regulators of the tumor microenvironment and immune evasion, with therapeutic potential. Cancers. (2025) 17:2172. doi: 10.3390/cancers17132172 40647470 PMC12248500

[17] Vaxevanis C Bachmann M Seliger B . Immune modulatory microRNAs in tumors, their clinical relevance in diagnosis and therapy. J Immunother Cancer. (2024) 12:e009774. doi: 10.1136/jitc-2024-009774 39209767 PMC11367391

[18] Omar H El-Serafi A Hersi F Arafa E Zaher D Madkour M . Immunomodulatory microRNAs in cancer: targeting immune checkpoints and the tumor microenvironment. FEBS J. (2019) 286:3540–57. doi: 10.1111/febs.15000 31306553

[19] Chavarriaga J Nappi L Papachristofilou A Conduit C Hamilton RJ . Testicular cancer. Lancet. (2025) 406:76–90. doi: 10.1016/s0140-6736(25)00455-6 40451233

[20] Bagrodia A Haugnes HS Hellesnes R Dabbas M Millard F Nappi L . Key updates in testicular cancer: Optimizing survivorship and survival. Am Soc Clin Oncol Educ Book. (2025) 45:e472654. doi: 10.1200/edbk-25-472654 40324110

[21] Patrikidou A Cazzaniga W Berney D Boormans J Angst I Nardo DD . European Association of Urology guidelines on testicular cancer: 2023 update. Eur Urol. (2023) 84:289–301. doi: 10.1016/j.eururo.2023.04.010 37183161

[22] Chahoud J Zhang M Shah A Lin S Pisters L Tu SM . Managing seminomatous and nonseminomatous germ cell tumors. Curr Opin Oncol. (2018) 30:181–8. doi: 10.1097/cco.0000000000000446 29538040

[23] Aguilar R Johnson J Barrett P Tuohy VK . Vaccination with inhibin-α provides effective immunotherapy against testicular stromal cell tumors. J Immunother Cancer. (2017) 5:37. doi: 10.1186/s40425-017-0237-2 28428886 PMC5394616

[24] Richardson N Adra N . Novel therapeutics in refractory germ cell tumors. Curr Opin Oncol. (2025) 37:267–73. doi: 10.1097/cco.0000000000001129 40065678

[25] Alahdal M Elkord E . Non-coding RNAs in cancer immunotherapy: Predictive biomarkers and targets. Clin Transl Med. (2023) 13:e1425. doi: 10.1002/ctm2.1425 37735815 PMC10514379

[26] Di MM Riillo C Scionti F Grillone K Polerà N Caracciolo D . miRNAs and lncRNAs as novel therapeutic targets to improve cancer immunotherapy. Cancers. (2021) 13:1587. doi: 10.3390/cancers13071587 33808190 PMC8036682

[27] Gomarasca M Maroni P Banfi G Lombardi G . microRNAs in the antitumor immune response and in bone metastasis of breast cancer: From biological mechanisms to therapeutics. Int J Mol Sci. (2020) 21:2805. doi: 10.3390/ijms21082805 32316552 PMC7216039

[28] Gramantieri L Fornari F Giovannini C Trerè D . MicroRNAs at the crossroad between immunoediting and oncogenic drivers in hepatocellular carcinoma. Biomolecules. (2022) 12:930. doi: 10.3390/biom12070930 35883486 PMC9313100

[29] Xing Y Wang Z Lu Z Xia J Xie Z Jiao M . MicroRNAs: immune modulators in cancer immunotherapy. Immunother Adv. (2021) 1:ltab006. doi: 10.1093/immadv/ltab006 35919742 PMC9327120

[30] Afra F Mahboobipour A Salehi FA Ala M . Recent progress in the immunotherapy of hepatocellular carcinoma: Non-coding RNA-based immunotherapy may improve the outcome. BioMed Pharmacother. (2023) 165:115104. doi: 10.1016/j.biopha.2023.115104 37393866

[31] Mastrolia I Catani V Oltrecolli M Pipitone S Vitale M Masciale V . Chasing the role of miRNAs in RCC: From free-circulating to extracellular-vesicle-derived biomarkers. Biology. (2023) 12:877. doi: 10.3390/biology12060877 37372161 PMC10295314

[32] Olejarz W Sadowski K Szulczyk D Basak G . Advancements in personalized CAR-T therapy: Comprehensive overview of biomarkers and therapeutic targets in hematological Malignancies. Int J Mol Sci. (2024) 25:7743. doi: 10.3390/ijms25147743 39062986 PMC11276786

[33] Greenbaum U Yalniz F Srour S Rezvani K Singh H Olson A . Chimeric antigen receptor therapy: How are we driving in solid tumors? Biol Blood Marrow Transplant. (2020) 26:1759–69. doi: 10.1016/j.bbmt.2020.06.020 32623078 PMC11409837

[34] Pote M Singh D M AA Suchita J Gacche RN . Cancer metastases: Tailoring the targets. Heliyon. (2024) 10:e35369. doi: 10.1016/j.heliyon.2024.e35369 39170575 PMC11336595

[35] Pawłowska A Rekowska A Kuryło W Pańczyszyn A Kotarski J Wertel I . Current understanding on why ovarian cancer is resistant to immune checkpoint inhibitors. Int J Mol Sci. (2023) 24:10859. doi: 10.3390/ijms241310859 37446039 PMC10341806

[36] AER K Prakash M Cox M Wilson K Boer J Cauchi J . Therapeutic cancer vaccines-T cell responses and epigenetic modulation. Front Immunol. (2019) 9:3109. doi: 10.3389/fimmu.2018.03109 30740111 PMC6357987

[37] Haque S Cook K Sahay G Sun C . RNA-based therapeutics: Current developments in targeted molecular therapy of triple-negative breast cancer. Pharmaceutics. (2021) 13:1694. doi: 10.3390/pharmaceutics13101694 34683988 PMC8537780

[38] Ma Y Wang T Zhang X Wang P Long F . The role of circular RNAs in regulating resistance to cancer immunotherapy: mechanisms and implications. Cell Death Dis. (2024) 15:312. doi: 10.1038/s41419-024-06698-3 38697964 PMC11066075

[39] Seyhan AA . Trials and tribulations of microRNA therapeutics. Int J Mol Sci. (2024) 25:1469. doi: 10.3390/ijms25031469 38338746 PMC10855871

[40] Nguyen H Guz-Montgomery K Lowe D Saha D . Pathogenetic features and current management of glioblastoma. Cancers. (2021) 13:856. doi: 10.3390/cancers13040856 33670551 PMC7922739

[41] Galanopoulos M Doukatas A Gkeros F Viazis N Liatsos C . Room for improvement in the treatment of pancreatic cancer: Novel opportunities from gene targeted therapy. World J Gastroenterol. (2021) 27:3568–80. doi: 10.3748/wjg.v27.i24.3568 34239270 PMC8240062

[42] Varrone F Caputo E . The miRNAs role in melanoma and in its resistance to therapy. Int J Mol Sci. (2020) 21:878. doi: 10.3390/ijms21030878 32013263 PMC7037367

[43] Saleh R Jasim S Kadhum W Hjazi A Faraz A Abid M . Exploring the detailed role of interleukins in cancer: A comprehensive review of literature. Pathol - Res Pr. (2024) 257:155284. doi: 10.1016/j.prp.2024.155284 38663179

[44] Chen J Jiang C Jin L Zhang XD . Regulation of PD-L1: a novel role of pro-survival signalling in cancer. Ann Oncol. (2016) 27:409–16. doi: 10.1093/annonc/mdv615 26681673

[45] Khan I Mahfooz S Elbasan E Karacam B Oztanir M Hatiboglu MA . Targeting glioblastoma: The current state of different therapeutic approaches. Curr Neuropharmacol. (2021) 19:1701–15. doi: 10.2174/1570159x19666210113152108 33441071 PMC8977637

[46] Lim S Boyd S Diefenbach R Rizos H . Circulating microRNAs: functional biomarkers for melanoma prognosis and treatment. Mol Cancer. (2025) 24:99. doi: 10.1186/s12943-025-02298-7 40156012 PMC11951542

[47] Dzobo K Senthebane D Ganz C Thomford N Wonkam A Dandara C . Advances in therapeutic targeting of cancer stem cells within the tumor microenvironment: An updated review. Cells. (2020) 9:1896. doi: 10.3390/cells9081896 32823711 PMC7464860

[48] Baskaran A Kozel O Venkatesh O Wainwright D Sonabend A Heimberger A . Immune checkpoint inhibitors in glioblastoma IDHwt treatment: A systematic review. Cancers. (2024) 16:4148. doi: 10.3390/cancers16244148 39766048 PMC11674442

[49] Meliante P Barbato C Zoccali F Ralli M Greco A Vincentiis M . Programmed cell death-ligand 1 in head and neck squamous cell carcinoma: Molecular insights, preclinical and clinical data, and therapies. Int J Mol Sci. (2022) 23:15384. doi: 10.3390/ijms232315384 36499710 PMC9738355

[50] Zielińska MK Ciążyńska M Sulejczak D Rutkowski P Czarnecka AM . Mechanisms of resistance to anti-PD-1 immunotherapy in melanoma and strategies to overcome it. Biomolecules. (2025) 15:269. doi: 10.3390/biom15020269 40001572 PMC11853485

[51] Ji H Zhang L Ye L . Exosome, an important transmitter in the drug resistance of non-small cell lung cancer. Front Oncol. (2025) 15:1539047. doi: 10.3389/fonc.2025.1539047 40444086 PMC12119617

[52] Luo Y Yang J Yu J Liu X Yu C Hu J . Long non-coding RNAs: Emerging roles in the immunosuppressive tumor microenvironment. Front Oncol. (2020) 10:48. doi: 10.3389/fonc.2020.00048 32083005 PMC7005925

[53] Tufail M Hu J Liang J He C Wan W Huang Y . Hallmarks of cancer resistance. iScience. (2024) 27:109979. doi: 10.1016/j.isci.2024.109979 38832007 PMC11145355

[54] Takeda Y Kobayashi S Kitakaze M Yamada D Akita H Asai A . Immuno-surgical management of pancreatic cancer with analysis of cancer exosomes. Cells. (2020) 9:1645. doi: 10.3390/cells9071645 32659892 PMC7408222

[55] Wang S Wang Z Liu M Sun X . Role of extracellular vesicles in cancer: Implications in immunotherapeutic resistance. Front Immunol. (2025) 16:1581635. doi: 10.3389/fimmu.2025.1581635 40475777 PMC12137360

[56] Zhou H Jia W Lu L Han R . MicroRNAs with multiple targets of immune checkpoints, as a potential sensitizer for immune checkpoint inhibitors in breast cancer treatment. Cancers. (2023) 15:824. doi: 10.3390/cancers15030824 36765782 PMC9913694

[57] Maqsood Q Khan M Fatima T Khalid S Malik ZI . Recent insights into breast cancer: Molecular pathways, epigenetic regulation, and emerging targeted therapies. Breast Cancer: Basic Clin Res. (2025) 19:11782234251355663. doi: 10.1177/11782234251355663 40661160 PMC12256763

[58] Schaible P Bethge W Lengerke C Haraszti RA . RNA therapeutics for improving CAR T-cell safety and efficacy. Cancer Res. (2023) 83:354–62. doi: 10.1158/0008-5472.can-22-2155 36512627 PMC7614194

[59] Liang H Zhou B Li P Zhang X Zhang S Zhang Y . Stemness regulation in prostate cancer: Prostate cancer stem cells and targeted therapy. Ann Med. (2025) 57:2442067. doi: 10.1080/07853890.2024.2442067 39711287 PMC11703425

[60] Niebora J Woźniak S Domagała D Data K Farzaneh M Zehtabi M . The role of ncRNAs and exosomes in the development and progression of endometrial cancer. Front Oncol. (2024) 14:1418005. doi: 10.3389/fonc.2024.1418005 39188680 PMC11345653

[61] Saha T Lukong KE . Breast cancer stem-like cells in drug resistance: A review of mechanisms and novel therapeutic strategies to overcome drug resistance. Front Oncol. (2022) 12:856974. doi: 10.3389/fonc.2022.856974 35392236 PMC8979779

[62] Manne A Woods E Tsung A Mittra A . Biliary tract cancers: Treatment updates and future directions in the era of precision medicine and immuno-oncology. Front Oncol. (2021) 11:768009. doi: 10.3389/fonc.2021.768009 34868996 PMC8634105

[63] Adra N Einhorn L Althouse S Ammakkanavar N Musapatika D Albany C . Phase II trial of pembrolizumab in patients with platinum refractory germ-cell tumors: A Hoosier Cancer Research Network Study GU14-206. Ann Oncol. (2018) 29:209–14. doi: 10.1093/annonc/mdx680 29045540

[64] Huang R Shu D Li H Hu A Chen M Yang W . Clinical outcome and prognostic factors for immunotherapy-based treatments in patients with platinum-refractory germ cell tumor. Int Immunopharmacol. (2024) 142:113042. doi: 10.1016/j.intimp.2024.113042 39236453

[65] Semaan A Haddad FG Eid R Kourie HR Nemr E . Immunotherapy: Last bullet in platinum refractory germ cell testicular cancer. Futur Oncol. (2019) 15:533–41. doi: 10.2217/fon-2018-0571 30624089

[66] Evmorfopoulos K Marsitopoulos K Karachalios R Karathanasis A Dimitropoulos K Tzortzis V . The immune landscape and immunotherapeutic strategies in platinum-refractory testicular germ cell tumors. Cancers. (2024) 16:428. doi: 10.3390/cancers16020428 38275869 PMC10814346

[67] Fankhauser CD Curioni-Fontecedro A Allmann V Beyer J Tischler V Sulser T . Frequent PD-L1 expression in testicular germ cell tumors. Br J Cancer. (2015) 113:411–3. doi: 10.1038/bjc.2015.244 26171934 PMC4522642

[68] Al-Hogbani M Duguay J Wagner D-C Haferkamp A Joubert P Frees S . Expression of programmed death ligand-1 (PD-L1) in metastatic and postchemotherapy viable testicular germ cell tumors. Urol Oncol Semin Orig Investig. (2021) 39:303.e1–8. doi: 10.1016/j.urolonc.2021.02.014 33685799

[69] Melotti S Ambrosi F Franceschini T Giunchi F Filippo D Franchini E . TAMs PD-L1(+) in the reprogramming of germ cell tumors of the testis. Pathol - Res Pr. (2023) 247:154540. doi: 10.1016/j.prp.2023.154540 37209574

[70] Farahani H Dehghanian AR Khademolhosseini A Haghshenas MR Erfani N . Frequent expression of CD45RO memory T cell marker as well as low to high expression of PD-1 and PD-L1 inhibitory molecules in seminoma and dysgerminoma. J Reprod Immunol. (2024) 161:104184. doi: 10.1016/j.jri.2023.104184 38171036

[71] Lobo J Rodrigues Â Guimarães R Cantante M Lopes P Maurício J . Detailed characterization of immune cell infiltrate and expression of immune checkpoint molecules PD-L1/CTLA-4 and MMR proteins in testicular germ cell tumors disclose novel disease biomarkers. Cancers. (2019) 11:1535. doi: 10.3390/cancers11101535 31614500 PMC6826711

[72] Klein B Haggeney T Fietz D Indumathy S Loveland KL Hedger M . Specific immune cell and cytokine characteristics of human testicular germ cell neoplasia. Hum Reprod. (2016) 31:2192–202. doi: 10.1093/humrep/dew211 27609978

[73] Klemke M Belge G Dieckmann K . Recent advances in understanding the biological role of microRNA‐371a‐3p in testicular germ cell tumors. Andrology. (2026):e70181. doi: 10.1111/andr.70181 41574394

[74] Islam R Heyer J Figura M Wang X Nie X Nathaniel B . T cells in testicular germ cell tumors: New evidence of fundamental contributions by rare subsets. Br J Cancer. (2024) 130:1893–903. doi: 10.1038/s41416-024-02669-9 38649788 PMC11183042

[75] Fijak M Meinhardt A . The testis in immune privilege. Immunol Rev. (2006) 213:66–81. doi: 10.1111/j.1600-065x.2006.00438.x 16972897

[76] Fijak M Bhushan S Meinhardt A . Suppression and regulation of immune responses, methods and protocols. Methods Mol Biol. (2010) 677:459–70. doi: 10.1007/978-1-60761-869-0_29 20941627

[77] U GS Madan R Aragon-Ching JB . The immunotherapy revolution in genitourinary Malignancies. Immunotherapy. (2020) 12:819–31. doi: 10.2217/imt-2020-0054 32594815 PMC7466927

[78] Oing C Bokemeyer C . Biological basis and early clinical results of immunotherapy for cisplatin-resistant germ cell cancer. Curr Opin Urol. (2018) 28:479–84. doi: 10.1097/mou.0000000000000531 29957683

[79] Bernard L . Treatment of recurrent ovarian germ cell tumors: Is there a role for immune checkpoint inhibitors? Gynecol Oncol Rep. (2024) 56:101502. doi: 10.1016/j.gore.2024.101502 39386925 PMC11460618

[80] Lafin J Bagrodia A Woldu S Amatruda JF . New insights into germ cell tumor genomics. Andrology. (2019) 7:507–15. doi: 10.1111/andr.12616 30896089

[81] Kalemoglu E Jani Y Canaslan K Bilen MA . The role of immunotherapy in targeting tumor microenvironment in genitourinary cancers. Front Immunol. (2025) 16:1506278. doi: 10.3389/fimmu.2025.1506278 40260236 PMC12009843

[82] Salazar-Mejía C Villarreal-González R Vidal-Gutiérrez O la C la C Guadarrama-Rendón E Alvarado-Ruiz S . Immune checkpoint inhibitors in patients with testicular cancer: A systematic review. J Adolesc Young Adult Oncol. (2025) 14:291–8. doi: 10.1089/jayao.2024.0056 39718949

[83] Kawahara T Kawai K Kojima T Nagumo Y Sakka S Kandori S . Phase II trial of nivolumab monotherapy and biomarker screening in patients with chemo-refractory germ cell tumors. Int J Urol. (2022) 29:741–7. doi: 10.1111/iju.14885 35462438 PMC9545636

[84] Zhang H Jiang D Meng E Zhao M Niu B . Long-term response to camrelizumab in a pretreated metastatic mixed testicular germ-cell tumor patient with co-mutations in DNA damage-repair genes. Immunotherapy. (2023) 15:17–25. doi: 10.2217/imt-2021-0259 36647794

[85] Országhová Z Kalavska K Mego M Chovanec M . Overcoming chemotherapy resistance in germ cell tumors. Biomedicines. (2022) 10:972. doi: 10.3390/biomedicines10050972 35625709 PMC9139090

[86] Laffi A Cozzi G Spada F Fazio N Bertuzzi A Santoro A . Metachronous testicular metastases from Merkel cell carcinoma (MCC): A case report and literature review. Am J Case Rep. (2022) 23:e936552-1-e936552-7. doi: 10.12659/ajcr.936552 36031755 PMC9438938

[87] Hong DS Kang Y-K Borad M Sachdev J Ejadi S Lim HY . Phase 1 study of MRX34, a liposomal miR-34a mimic, in patients with advanced solid tumors. Br J Cancer. (2020) 122:1630–7. doi: 10.1038/s41416-020-0802-1 32238921 PMC7251107

[88] Beg MS Brenner AJ Sachdev J Borad M Kang Y-K Stoudemire J . Phase I study of MRX34, a liposomal miR-34a mimic, administered twice weekly in patients with advanced solid tumors. Investig N Drugs. (2017) 35:180–8. doi: 10.1007/s10637-016-0407-y 27917453 PMC5893501

[89] Szczepanek J Skorupa M Tretyn A . MicroRNA as a potential therapeutic molecule in cancer. Cells. (2022) 11:1008. doi: 10.3390/cells11061008 35326459 PMC8947269

[90] Elesawy A Abulsoud A HAM M Elballal M Sallam A Elazazy O . miRNAs orchestration of testicular germ cell tumors - Particular emphasis on diagnosis, progression and drug resistance. Pathol - Res Pr. (2023) 248:154612. doi: 10.1016/j.prp.2023.154612 37327566

[91] Voorhoeve PM Sage C Schrier M Gillis AJM Stoop H Nagel R . A genetic screen implicates miRNA-372 and miRNA-373 as oncogenes in testicular germ cell tumors. Cell. (2006) 124:1169–81. doi: 10.1016/j.cell.2006.02.037 16564011

[92] Zhou A-D Diao L-T Xu H Xiao Z-D Li J-H Zhou H . β-catenin/LEF1 transactivates the microRNA-371–373 cluster that modulates the Wnt/β-catenin-signaling pathway. Oncogene. (2012) 31:2968–78. doi: 10.1038/onc.2011.461 22020335

[93] Belge G Dieckmann K-P Spiekermann M Balks T Bullerdiek J . Serum levels of microRNAs miR-371-3: A novel class of serum biomarkers for testicular germ cell tumors? Eur Urol. (2012) 61:1068–9. doi: 10.1016/j.eururo.2012.02.037 22386195

[94] Regouc M Belge G Lorch A Dieckmann K-P Pichler M . Non-coding microRNAs as novel potential tumor markers in testicular cancer. Cancers. (2020) 12:749. doi: 10.3390/cancers12030749 32235691 PMC7140096

[95] Batool A Liu X-M Zhang C-L Hao C-F Chen S-R Liu Y-X . Recent advances in the regulation of testicular germ cell tumors by microRNAs. Front Biosci. (2019) 24:765–76. doi: 10.2741/4749 30844711

[96] Lobo J Gillis AJM Berg A Dorssers LCJ Belge G Dieckmann K-P . Identification and validation model for informative liquid biopsy-based microRNA biomarkers: insights from germ cell tumor *In Vitro*, *In Vivo* and patient-derived data. Cells. (2019) 8:1637. doi: 10.3390/cells8121637 31847394 PMC6952794

[97] Agthoven T Looijenga LHJ . Accurate primary germ cell cancer diagnosis using serum based microRNA detection (ampTSmiR test). Oncotarget. (2016) 8:58037–49. doi: 10.18632/oncotarget.10867 28938535 PMC5601631

[98] Syring I Bartels J Holdenrieder S Kristiansen G Müller SC Ellinger J . Circulating serum miRNA (miR-367-3p, miR-371a-3p, miR-372-3p and miR-373-3p) as biomarkers in patients with testicular germ cell cancer. J Urol. (2015) 193:331–7. doi: 10.1016/j.juro.2014.07.010 25046619

[99] Dieckmann K-P Radtke A Spiekermann M Balks T Matthies C Becker P . Serum levels of microRNA miR-371a-3p: a sensitive and specific new biomarker for germ cell tumors. Eur Urol. (2017) 71:213–20. doi: 10.1016/j.eururo.2016.07.029 27495845

[100] Radtke A Cremers J-F Kliesch S Riek S Junker K Mohamed SA . Can germ cell neoplasia in situ be diagnosed by measuring serum levels of microRNA371a-3p? J Cancer Res Clin Oncol. (2017) 143:2383–92. doi: 10.1007/s00432-017-2490-7 28819887 PMC5640733

[101] Ditonno F Franco A Manfredi C Fasanella D Abate M Rocca RL . The role of miRNA in testicular cancer: current insights and future perspectives. Medicina. (2023) 59:2033. doi: 10.3390/medicina59112033 38004082 PMC10672751

[102] Dieckmann K-P Radtke A Geczi L Matthies C Anheuser P Eckardt U . Serum levels of microRNA-371a-3p (M371 test) as a new biomarker of testicular germ cell tumors: results of a prospective multicentric study. J Clin Oncol. (2019) 37:JCO.18.01480. doi: 10.1200/jco.18.01480 30875280 PMC6544462

[103] Terbuch A Adiprasito J Stiegelbauer V Seles M Klec C Pichler G . MiR-371a-3p serum levels are increased in recurrence of testicular germ cell tumor patients. Int J Mol Sci. (2018) 19:3130. doi: 10.3390/ijms19103130 30321995 PMC6213366

[104] Mego M Agthoven T Gronesova P Chovanec M Miskovska V Mardiak J . Clinical utility of plasma miR‐371a‐3p in germ cell tumors. J Cell Mol Med. (2019) 23:1128–36. doi: 10.1111/jcmm.14013 30536846 PMC6349199

[105] Bezan A Gerger A Pichler M . MicroRNAs in testicular cancer: implications for pathogenesis, diagnosis, prognosis and therapy. Anticancer Res. (2014) 34:2709–13. 24922631

[106] Nappi L Thi M Lum A Huntsman D Eigl BJ Martin C . Developing a highly specific biomarker for germ cell Malignancies: plasma miR371 expression across the germ cell Malignancy spectrum. J Clin Oncol. (2019) 37:3090–8. doi: 10.1200/jco.18.02057 31553692 PMC7351323

[107] Badia RR Abe D Wong D Singla N Savelyeva A Chertack N . Real-world application of pre-orchiectomy miR-371a-3p test in testicular germ cell tumor management. J Urol. (2021) 205:137–44. doi: 10.1097/ju.0000000000001337 32856980

[108] Lafin JT Singla N Woldu SL Lotan Y Lewis CM Majmudar K . Serum microRNA-371a-3p levels predict viable germ cell tumor in chemotherapy-naïve patients undergoing retroperitoneal lymph node dissection. Eur Urol. (2020) 77:290–2. doi: 10.1016/j.eururo.2019.10.005 31699528 PMC7756387

[109] Vilela-Salgueiro B Barros-Silva D Lobo J Costa AL Guimarães R Cantante M . Germ cell tumor subtypes display differential expression of microRNA371a-3p. Philos Trans R Soc B Biol Sci. (2018) 373:20170338. doi: 10.1098/rstb.2017.0338 29685967 PMC5915726

[110] Dieckmann K-P Hennig F Anheuser P Gehrckens R Viehweger F Wülfing C . High expression of microRNA-371a-3p in cystic fluid of post-chemotherapy teratoma with concurrent normal serum levels in patients with non-seminomatous testicular germ cell tumors. Urol Int. (2021) 105:21–6. doi: 10.1159/000510760 33049748

[111] Nappi L Thi M Adra N Hamilton RJ Leao R Lavoie J-M . Integrated expression of circulating miR375 and miR371 to identify teratoma and active germ cell Malignancy components in Malignant germ cell tumors. Eur Urol. (2021) 79:16–9. doi: 10.1016/j.eururo.2020.10.024 33158661

[112] Lobo J Jerónimo C Henrique R . Cisplatin resistance in testicular germ cell tumors: current challenges from various perspectives. Cancers. (2020) 12:1601. doi: 10.3390/cancers12061601 32560427 PMC7352163

[113] Weiten R Engler T Schorle H Ellinger J Saponaro M Alajati A . The new tumor biomarker miRNA‐371‐3p influences cisplatin sensitivity of testicular germ cell tumor cell lines. J Cell Mol Med. (2024) 28:e70314. doi: 10.1111/jcmm.70314 39706819 PMC11661915

[114] Nettersheim D Arndt I Sharma R Riesenberg S Jostes S Schneider S . The cancer/testis-antigen PRAME supports the pluripotency network and represses somatic and germ cell differentiation programs in seminomas. Br J Cancer. (2016) 115:454–64. doi: 10.1038/bjc.2016.187 27441500 PMC4985348

[115] Wang X Li J Dong K Lin F Long M Ouyang Y . Tumor suppressor miR-34a targets PD-L1 and functions as a potential immunotherapeutic target in acute myeloid leukemia. Cell Signal. (2015) 27:443–52. doi: 10.1016/j.cellsig.2014.12.003 25499621

[116] Mazurek M Litak J Kamieniak P Osuchowska I Maciejewski R Roliński J . Micro RNA molecules as modulators of treatment resistance, immune checkpoints controllers and sensitive biomarkers in glioblastoma multiforme. Int J Mol Sci. (2020) 21:1507. doi: 10.3390/ijms21041507 32098401 PMC7073212

[117] Wilczyński M Wilczyński J Nowak M . MiRNAs as regulators of immune cells in the tumor microenvironment of ovarian cancer. Cells. (2024) 13:1343. doi: 10.3390/cells13161343 39195233 PMC11352322

[118] Alotaibi F . Exosomal microRNAs in cancer: potential biomarkers and immunotherapeutic targets for immune checkpoint molecules. Front Genet. (2023) 14:1052731. doi: 10.3389/fgene.2023.1052731 36873941 PMC9982116

[119] Angerilli V Galuppini F Businello G Dal SL Savarino E Realdon S . MicroRNAs as predictive biomarkers of resistance to targeted therapies in gastrointestinal tumors. Biomedicines. (2021) 9:318. doi: 10.3390/biomedicines9030318 33801049 PMC8003870

[120] Dong L Tian X Zhao Y Tu H Wong A Yang Y . The roles of miRNAs (microRNAs) in melanoma immunotherapy. Int J Mol Sci. (2022) 23:14775. doi: 10.3390/ijms232314775 36499102 PMC9736803

[121] Kousar K Ahmad T Abduh M Kanwal B Shah S Naseer F . miRNAs in regulation of tumor microenvironment, chemotherapy resistance, immunotherapy modulation and miRNA therapeutics in cancer. Int J Mol Sci. (2022) 23:13822. doi: 10.3390/ijms232213822 36430305 PMC9699074

[122] Zhu J Chen L Zou L Yang P Wu R Mao Y . MiR-20b, -21, and -130b inhibit PTEN expression resulting in B7-H1 over-expression in advanced colorectal cancer. Hum Immunol. (2014) 75:348–53. doi: 10.1016/j.humimm.2014.01.006 24468585

[123] Zhao Y Shen M Wu L Yang H Yao Y Yang Q . Stromal cells in the tumor microenvironment: accomplices of tumor progression? Cell Death Dis. (2023) 14:587. doi: 10.1038/s41419-023-06110-6 37666813 PMC10477351

[124] Mao X Xu J Wang W Liang C Hua J Liu J . Crosstalk between cancer-associated fibroblasts and immune cells in the tumor microenvironment: new findings and future perspectives. Mol Cancer. (2021) 20:131. doi: 10.1186/s12943-021-01428-1 34635121 PMC8504100

[125] Lasser SA Kurt FGO Arkhypov I Utikal J Umansky V . Myeloid-derived suppressor cells in cancer and cancer therapy. Nat Rev Clin Oncol. (2024) 21:147–64. doi: 10.1038/s41571-023-00846-y 38191922

[126] Gabrilovich DI Nagaraj S . Myeloid-derived suppressor cells as regulators of the immune system. Nat Rev Immunol. (2009) 9:162–74. doi: 10.1038/nri2506 19197294 PMC2828349

[127] Chen J Ye Y Liu P Yu W Wei F Li H . Suppression of T cells by myeloid-derived suppressor cells in cancer. Hum Immunol. (2017) 78:113–9. doi: 10.1016/j.humimm.2016.12.001 27939507

[128] Huber V Vallacchi V Fleming V Hu X Cova A Dugo M . Tumor-derived microRNAs induce myeloid suppressor cells and predict immunotherapy resistance in melanoma. J Clin Investig. (2018) 128:5505–16. doi: 10.1172/jci98060 30260323 PMC6264733

[129] Arghiani N Shah K . Modulating microRNAs in cancer: next-generation therapies. Cancer Biol Med. (2021) 19:289–304. doi: 10.20892/j.issn.2095-3941.2021.0294 34846108 PMC8958885

[130] Chen S Zhang Y Kuzel TM Zhang B . Regulating tumor myeloid-derived suppressor cells by microRNAs. Cancer Cell Microenviron. (2015) 2(1):e637. doi: 10.14800/ccm.637 26005707 PMC4440580

[131] Huffaker TB Lee S-H Tang WW Wallace JA Alexander M Runtsch MC . Antitumor immunity is defective in T cell–specific microRNA-155–deficient mice and is rescued by immune checkpoint blockade. J Biol Chem. (2017) 292:18530–41. doi: 10.1074/jbc.m117.808121 28912267 PMC5682963

[132] Yin Y Cai X Chen X Liang H Zhang Y Li J . Tumor-secreted miR-214 induces regulatory T cells: a major link between immune evasion and tumor growth. Cell Res. (2014) 24:1164–80. doi: 10.1038/cr.2014.121 25223704 PMC4185347

[133] Khalaf K Hana D Chou J Singh C Mackiewicz A Kaczmarek M . Aspects of the tumor microenvironment involved in immune resistance and drug resistance. Front Immunol. (2021) 12:656364. doi: 10.3389/fimmu.2021.656364 34122412 PMC8190405

[134] Nguyen M Luo Y Li A Tsai J Wu K Chung P . miRNA as a modulator of immunotherapy and immune response in melanoma. Biomolecules. (2021) 11:1648. doi: 10.3390/biom11111648 34827646 PMC8615556

[135] Parayath NN Gandham SK Leslie F Amiji MM . Improved anti-tumor efficacy of paclitaxel in combination with microRNA-125b-based tumor-associated macrophage repolarization in epithelial ovarian cancer. Cancer Lett. (2019) 461:1–9. doi: 10.1016/j.canlet.2019.07.002 31288064 PMC6682447

[136] Gerloff D Lützkendorf J Moritz RKC Wersig T Mäder K Müller LP . Melanoma-derived exosomal miR-125b-5p educates tumor associated macrophages (TAMs) by targeting lysosomal acid lipase A (LIPA). Cancers. (2020) 12:464. doi: 10.3390/cancers12020464 32079286 PMC7072270

[137] Li Z Suo B Long G Gao Y Song J Zhang M . Exosomal miRNA-16-5p derived from M1 macrophages enhances T cell-dependent immune response by regulating PD-L1 in gastric cancer. Front Cell Dev Biol. (2020) 8:572689. doi: 10.3389/fcell.2020.572689 33330451 PMC7734296

[138] Weng Y-S Tseng H-Y Chen Y-A Shen P-C Haq ATA Chen L-M . MCT-1/miR-34a/IL-6/IL-6R signaling axis promotes EMT progression, cancer stemness and M2 macrophage polarization in triple-negative breast cancer. Mol Cancer. (2019) 18:42. doi: 10.1186/s12943-019-0988-0 30885232 PMC6421700

[139] Tung SL Boardman DA Sen M Letizia M Peng Q Cianci N . Regulatory T cell-derived extracellular vesicles modify dendritic cell function. Sci Rep. (2018) 8:6065. doi: 10.1038/s41598-018-24531-8 29666503 PMC5904112

[140] Zhou M Chen J Zhou L Chen W Ding G Cao L . Pancreatic cancer derived exosomes regulate the expression of TLR4 in dendritic cells via miR-203. Cell Immunol. (2014) 292:65–9. doi: 10.1016/j.cellimm.2014.09.004 25290620

[141] Berchem G Noman MZ Bosseler M Paggetti J Baconnais S cam EL . Hypoxic tumor-derived microvesicles negatively regulate NK cell function by a mechanism involving TGF-β and miR23a transfer. Oncoimmunology. (2015) 5:e1062968. doi: 10.1080/2162402x.2015.1062968 27141372 PMC4839360

[142] Heinemann A Zhao F Pechlivanis S Eberle J Steinle A Diederichs S . Tumor suppressive microRNAs miR-34a/c control cancer cell expression of ULBP2, a stress-induced ligand of the natural killer cell receptor NKG2D. Cancer Res. (2012) 72:460–71. doi: 10.1158/0008-5472.can-11-1977 22102694

[143] Tsukerman P Stern-Ginossar N Gur C Glasner A Nachmani D Bauman Y . MiR-10b downregulates the stress-induced cell surface molecule MICB, a critical ligand for cancer cell recognition by natural killer cells. Cancer Res. (2012) 72:5463–72. doi: 10.1158/0008-5472.can-11-2671 22915757

[144] Zhou Y Ren H Dai B Li J Shang L Huang J . Hepatocellular carcinoma-derived exosomal miRNA-21 contributes to tumor progression by converting hepatocyte stellate cells to cancer-associated fibroblasts. J Exp Clin Cancer Res CR. (2018) 37:324. doi: 10.1186/s13046-018-0965-2 30591064 PMC6307162

[145] Lin F Yin H-B Li X-Y Zhu G-M He W-Y Gou X . Bladder cancer cell-secreted exosomal miR-21 activates the PI3K/AKT pathway in macrophages to promote cancer progression. Int J Oncol. (2019) 56:151–64. doi: 10.3892/ijo.2019.4933 31814034 PMC6910194

[146] Chen H Luo Y Lin M Peng X Liu M Wang Y . Serum exosomal miR‐16‐5p functions as a tumor inhibitor and a new biomarker for PD‐L1 inhibitor‐dependent immunotherapy in lung adenocarcinoma by regulating PD‐L1 expression. Cancer Med. (2022) 11:2627–43. doi: 10.1002/cam4.4638 35347894 PMC9249988

[147] Zhang J Zhu J Zheng G Wang Q Li X Feng Y . Co-expression of miR155 or LSD1 shRNA increases the anti-tumor functions of CD19 CAR-T cells. Front Immunol. (2022) 12:811364. doi: 10.3389/fimmu.2021.811364 35046962 PMC8761951

[148] Huang Q Xia J Wang L Wang X Ma X Deng Q . miR-153 suppresses IDO1 expression and enhances CAR T cell immunotherapy. J Hematol Oncol. (2018) 11:58. doi: 10.1186/s13045-018-0600-x 29685162 PMC5914051

[149] Marschner D Falk M Javorniczky NR Hanke-Müller K Rawluk J Schmitt-Graeff A . MicroRNA-146a regulates immune-related adverse events caused by immune checkpoint inhibitors. JCI Insight. (2020) 5(6):e132334. doi: 10.1172/jci.insight.132334 32125286 PMC7213806

[150] Querfeld C Foss FM Kim YH Pinter-Brown L William BM Porcu P . Phase 1 trial of cobomarsen, an inhibitor of Mir-155, in cutaneous T cell lymphoma. Blood. (2018) 132:2903. doi: 10.1182/blood-2018-99-119861

[151] El-Daly S Bayraktar R Anfossi S Calin GA . The interplay between microRNAs and the components of the tumor microenvironment in B-cell Malignancies. Int J Mol Sci. (2020) 21:3387. doi: 10.3390/ijms21093387 32403283 PMC7246984

[152] Ukai M Yokoi A Yoshida K Suzuki S Shibata K Kikkawa F . Extracellular miRNAs as predictive biomarkers for glypican-3-derived peptide vaccine therapy response in ovarian clear cell carcinoma. Cancers. (2021) 13:550. doi: 10.3390/cancers13030550 33535558 PMC7867082

[153] Kijima T Hazama S Tsunedomi R Tanaka H Takenouchi H Kanekiyo S . MicroRNA-6826 and -6875 in plasma are valuable non-invasive biomarkers that predict the efficacy of vaccine treatment against metastatic colorectal cancer. Oncol Rep. (2016) 37:23–30. doi: 10.3892/or.2016.5267 27878288 PMC5355687

[154] Hodge J Wang F Wang J Liu Q Saaoud F Wang Y . Overexpression of microRNA-155 enhances the efficacy of dendritic cell vaccine against breast cancer. OncoImmunology. (2020) 9:1724761. doi: 10.1080/2162402x.2020.1724761 32117588 PMC7028336

[155] Lizé M Pilarski S Dobbelstein M . E2F1-inducible microRNA 449a/b suppresses cell proliferation and promotes apoptosis. Cell Death Differ. (2010) 17:452–8. doi: 10.1038/cdd.2009.188 19960022

[156] Martino MD Chieffi P Esposito F . miRNAs and biomarkers in testicular germ cell tumors: An update. Int J Mol Sci. (2021) 22:1380. doi: 10.3390/ijms22031380 33573132 PMC7866514

[157] Li Y Zhou T Cheng X Li D Zhao M Zheng WV . microRNA-378a-3p regulates the progression of hepatocellular carcinoma by regulating PD-L1 and STAT3. Bioengineered. (2022) 13:4730–43. doi: 10.1080/21655979.2022.2031408 35184646 PMC8973785

[158] Cortez MA Valdecanas D Wang X Ivan C Peltier H Ye H . Abstract 2875: p53 regulation of PDL1 is mediated through miR-34a. Cancer Res. (2015) 75:2875. doi: 10.1158/1538-7445.am2015-2875 26062558

[159] Chang T-C Wentzel EA Kent OA Ramachandran K Mullendore M Lee KH . Transactivation of miR-34a by p53 broadly influences gene expression and promotes apoptosis. Mol Cell. (2007) 26:745–52. doi: 10.1016/j.molcel.2007.05.010 17540599 PMC1939978

[160] Cortez MA Ivan C Valdecanas D Wang X Peltier HJ Ye Y . PDL1 regulation by p53 via miR-34. JNCI J Natl Cancer Inst. (2015) 108:djv303. doi: 10.1093/jnci/djv303 26577528 PMC4862407

[161] Huemer F Leisch M Geisberger R Zaborsky N Greil R . miRNA-based therapeutics in the era of immune-checkpoint inhibitors. Pharmaceuticals. (2021) 14:89. doi: 10.3390/ph14020089 33530393 PMC7911012

[162] Shadbad M Safaei S Brunetti O Derakhshani A Lotfinejad P Mokhtarzadeh A . A systematic review on the therapeutic potentiality of PD-L1-inhibiting microRNAs for triple-negative breast cancer: Toward single-cell sequencing-guided biomimetic delivery. Genes. (2021) 12:1206. doi: 10.3390/genes12081206 34440380 PMC8391239

[163] Pan W Chai B Li L Lu Z Ma Z . p53/microRNA-34 axis in cancer and beyond. Heliyon. (2023) 9:e15155. doi: 10.1016/j.heliyon.2023.e15155 37095919 PMC10121403

[164] Kawai K Tawada A Onozawa M Inoue T Sakurai H Mori I . Rapid response to pembrolizumab in a chemo-refractory testicular germ cell cancer with microsatellite instability-high. OncoTargets Ther. (2021) 14:4853–8. doi: 10.2147/ott.s323898 34584425 PMC8464369

[165] Zhu S Wang Y Tang J Cao M . Radiotherapy induced immunogenic cell death by remodeling tumor immune microenvironment. Front Immunol. (2022) 13:1074477. doi: 10.3389/fimmu.2022.1074477 36532071 PMC9753984

[166] Shokry D Khan MW Powell C Johnson S Rennels BC Boyd RI . Refractory testicular germ cell tumors are highly sensitive to the targeting of polycomb pathway demethylases KDM6A and KDM6B. Cell Commun Signal. (2024) 22:528. doi: 10.1186/s12964-024-01912-3 39482699 PMC11529429

[167] Albany C Hever-Jardine MP Herrmann K Yim CY Tam J Warzecha JM . Refractory testicular germ cell tumors are highly sensitive to the second generation DNA methylation inhibitor guadecitabine. Oncotarget. (2016) 8:2949–59. doi: 10.18632/oncotarget.13811 27936464 PMC5356854

[168] Karikó K Buckstein M Ni H Weissman D . Suppression of RNA recognition by Toll-like receptors: The impact of nucleoside modification and the evolutionary origin of RNA. Immunity. (2005) 23:165–75. doi: 10.1016/j.immuni.2005.06.008 16111635

[169] Anderson BR Muramatsu H Jha BK Silverman RH Weissman D Karikó K . Nucleoside modifications in RNA limit activation of 2′-5′-oligoadenylate synthetase and increase resistance to cleavage by RNase L. Nucleic Acids Res. (2011) 39:9329–38. doi: 10.1093/nar/gkr586 21813458 PMC3241635

[170] Abdelaal AM Sohal IS Iyer S Sudarshan K Kothandaraman H Lanman NA . A first-in-class fully modified version of miR-34a with outstanding stability, activity, and anti-tumor efficacy. Oncogene. (2023) 42:2985–99. doi: 10.1038/s41388-023-02801-8 37666938 PMC10541324

[171] Fang L-L Wang X-H Sun B-F Zhang X-D Zhu X-H Yu Z-J . Expression, regulation and mechanism of action of the miR-17–92 cluster in tumor cells (Review). Int J Mol Med. (2017) 40:1624–30. doi: 10.3892/ijmm.2017.3164 29039606 PMC5716450

[172] Mogilyansky E Rigoutsos I . The miR-17/92 cluster: a comprehensive update on its genomics, genetics, functions and increasingly important and numerous roles in health and disease. Cell Death Differ. (2013) 20:1603–14. doi: 10.1038/cdd.2013.125 24212931 PMC3824591

[173] Novotny GW Sonne SB Nielsen JE Jonstrup SP Hansen MA Skakkebaek NE . Translational repression of E2F1 mRNA in carcinoma in situ and normal testis correlates with expression of the miR-17–92 cluster. Cell Death Differ. (2007) 14:879–82. doi: 10.1038/sj.cdd.4402090 17218954

[174] Sun W Cui J Ge Y Wang J Yu Y Han B . Tumor stem cell-derived exosomal microRNA-17-5p inhibits anti-tumor immunity in colorectal cancer via targeting SPOP and overexpressing PD-L1. Cell Death Discov. (2022) 8:223. doi: 10.1038/s41420-022-00919-4 35461336 PMC9035163

[175] Fontana L Fiori ME Albini S Cifaldi L Giovinazzi S Forloni M . Antagomir-17-5p abolishes the growth of therapy-resistant neuroblastoma through p21 and BIM. PloS One. (2008) 3:e2236. doi: 10.1371/journal.pone.0002236 18493594 PMC2375057

[176] Schena FP Pasculli E . RNA therapeutics in kidney diseases: prospects and current status. Clin Kidney J. (2025) 18:sfaf214. doi: 10.1093/ckj/sfaf214 40755967 PMC12314272

[177] trials.gov C . A study of RGLS4326 in patients with autosomal dominant polycystic kidney disease (2021). Available online at: https://clinicaltrials.gov/study/NCT04536688?utm_source=chatgpt.com#collaborators-and-investigators (Accessed May 20, 2026).

[178] Yu ASL Garg R Bellovich KA Silva AL Padgett CS Lee ECY . RGLS8429 increases urinary PC1 and PC2 and may reduce height-adjusted total kidney volume (htTKV) in patients with ADPKD. J Am Soc Nephrol. (2024) 35:10.1681/ASN.2024w1ps7ssb. doi: 10.1681/asn.2024w1ps7ssb

[179] Valencia T Yen LY Berman C Vincent T Davis S Varrone F . The nucleobase guanine at the 3’-terminus of oligonucleotide RGLS4326 drives off-target AMPAR inhibition and CNS toxicity. Nat Commun. (2025) 16:10762. doi: 10.1038/s41467-025-65799-5 41315228 PMC12663328

[180] Koster R Vugt M Timmer-Bosscha H Gietema JA Jong S . Unravelling mechanisms of cisplatin sensitivity and resistance in testicular cancer. Expert Rev Mol Med. (2013) 15:e12. doi: 10.1017/erm.2013.13 24074238

[181] Koster R Pietro A Timmer-Bosscha H Gibcus JH Berg A Suurmeijer AJ . Cytoplasmic p21 expression levels determine cisplatin resistance in human testicular cancer. J Clin Investig. (2010) 120:3594–605. doi: 10.1172/jci41939 20811155 PMC2947220

[182] Zhang Z Liu X Chen D Yu J . Radiotherapy combined with immunotherapy: the dawn of cancer treatment. Signal Transduct Target Ther. (2022) 7:258. doi: 10.1038/s41392-022-01102-y 35906199 PMC9338328

[183] Tesniere A Schlemmer F Boige V Kepp O Martins I Ghiringhelli F . Immunogenic death of colon cancer cells treated with oxaliplatin. Oncogene. (2010) 29:482–91. doi: 10.1038/onc.2009.356 19881547

[184] Ribas A Medina T Kummar S Amin A Kalbasi A Drabick JJ . SD-101 in combination with pembrolizumab in advanced melanoma: Results of a phase Ib, multicenter study. Cancer Discov. (2018) 8:1250–7. doi: 10.1158/2159-8290.cd-18-0280 30154193 PMC6719557

[185] Shapouri F Saeidi S Kakhki SA Pouyan O Amirchaghmaghi E Aflatoonian R . The expression of Toll-Like Receptors (TLRs) in testicular cancer: A case control study. Iran J Reprod Med. (2013) 11:919–24. PMC3941388 24639717

[186] Meric-Bernstam F Sweis RF Kasper S Hamid O Bhatia S Dummer R . Combination of the STING agonist MIW815 (ADU-S100) and PD-1 inhibitor spartalizumab in advanced/metastatic solid tumors or lymphomas: An open-label, multicenter, phase Ib study. Clin Cancer Res. (2022) 29:110–21. doi: 10.1158/1078-0432.ccr-22-2235 36282874 PMC11188043

[187] Ran X Wu BX Vidhyasagar V Song L Zhang X Ladak RJ . PARP inhibitor radiosensitization enhances anti-PD-L1 immunotherapy through stabilizing chemokine mRNA in small cell lung cancer. Nat Commun. (2025) 16:2166. doi: 10.1038/s41467-025-57257-z 40038278 PMC11880360

[188] Parola S Oing C Rescigno P Feliciano S Carlino F Pompella L . PARP inhibitors in testicular germ cell tumors: what we know and what we are looking for. Front Genet. (2024) 15:1480417. doi: 10.3389/fgene.2024.1480417 39678373 PMC11638157

[189] Roška J Lobo J Ivovič D Wachsmannová L Mueller T Henrique R . Integrated microarray-based data analysis of miRNA expression profiles: Identification of novel biomarkers of cisplatin-resistance in testicular germ cell tumors. Int J Mol Sci. (2023) 24:2495. doi: 10.3390/ijms24032495 36768818 PMC9916636

[190] Xu Z Yao T Liu W . miR-378a-3p sensitizes ovarian cancer cells to cisplatin through targeting MAPK1/GRB2. BioMed Pharmacother. (2018) 107:1410–7. doi: 10.1016/j.biopha.2018.08.132 30257357

[191] Castellani G Buccarelli M Lulli V Ilari R Luca DG Pedini F . Mir-378a-3p acts as a tumor suppressor in colorectal cancer stem-like cells and affects the expression of MALAT1 and NEAT1 lncRNAs. Front Oncol. (2022) 12:867886. doi: 10.3389/fonc.2022.867886 35814429 PMC9263271

[192] Albany C Fazal Z Singh R Bikorimana E Adra N Hanna NH . A phase 1 study of combined guadecitabine and cisplatin in platinum refractory germ cell cancer. Cancer Med. (2020) 10:156–63. doi: 10.1002/cam4.3583 33135391 PMC7826483

[193] Balaraman AK Babu MA Afzal M Sanghvi G M RM Gupta S . Exosome-based miRNA delivery: Transforming cancer treatment with mesenchymal stem cells. Regener Ther. (2025) 28:558–72. doi: 10.1016/j.reth.2025.01.019 40034540 PMC11872554

[194] Babaei S Fadaee M Abbasi-kenarsari H Shanehbandi D Kazemi T . Exosome-based immunotherapy as an innovative therapeutic approach in melanoma. Cell Commun Signal. (2024) 22:527. doi: 10.1186/s12964-024-01906-1 39482766 PMC11526674

[195] Chen J Hu S Liu J Jiang H Wang S Yang Z . Exosomes: a double‐edged sword in cancer immunotherapy. MedComm. (2025) 6:e70095. doi: 10.1002/mco2.70095 39968497 PMC11831209

[196] Lou Y Wang Y Lu J Chen X . Microrna-targeted nanoparticle delivery systems for cancer therapy: current status and future prospects. Nanomedicine. (2025) 20:1181–94. doi: 10.1080/17435889.2025.2492542 40231694 PMC12068351

[197] Han HD Mora EM Roh JW Nishimura M Lee SJ Stone RL . Chitosan hydrogel for localized gene silencing. Cancer Biol Ther. (2011) 11:839–45. doi: 10.4161/cbt.11.9.15185 21358280 PMC3100632

[198] Conde J Oliva N Atilano M Song HS Artzi N . Self-assembled RNA-triple-helix hydrogel scaffold for microrna modulation in the tumor microenvironment. Nat Mater. (2016) 15:353–63. doi: 10.1038/nmat4497 26641016 PMC6594154

[199] Zhong R Talebian S Mendes BB Wallace G Langer R Conde J . Hydrogels for RNA delivery. Nat Mater. (2023) 22:818–31. doi: 10.1038/s41563-023-01472-w 36941391 PMC10330049

[200] Xu H Fei Y Wang X Jiao W Jin Y . Advances in hydrogel-based delivery of RNA drugs for antitumor therapy. Gels. (2025) 11:633. doi: 10.3390/gels11080633 40868764 PMC12385893

[201] Liu X Zhou Q Yang Y Chen E . Application of hydrogels in cancer immunotherapy: a bibliometric analysis. Front Immunol. (2024) 15:1433050. doi: 10.3389/fimmu.2024.1433050 39192983 PMC11347446

[202] Adra N Vaughn DJ Einhorn LH Hanna NH Funt SA Rosales M . A phase II study assessing the safety and efficacy of ASP1650 in male patients with relapsed refractory germ cell tumors. Investig N Drugs. (2022) 40:1087–94. doi: 10.1007/s10637-022-01276-w 35759134 PMC10207925

[203] Skowron MA Kotthoff M Bremmer F Ruhnke K Parmaksiz F Richter A . Targeting CLDN6 in germ cell tumors by an antibody-drug-conjugate and studying therapy resistance of yolk-sac tumors to identify and screen specific therapeutic options. Mol Med. (2023) 29:40. doi: 10.1186/s10020-023-00636-3 36991316 PMC10053054

[204] Gillis A Stoop H Hersmus R Oosterhuis J Sun Y Chen C . High‐throughput microRNAome analysis in human germ cell tumors. J Pathol. (2007) 213:319–28. doi: 10.1002/path.2230 17893849

[205] Wang X Luo G Zhang K Cao J Huang C Jiang T . Hypoxic tumor-derived exosomal miR-301a mediates M2 macrophage polarization via PTEN/PI3Kγ to promote pancreatic cancer metastasis. Cancer Res. (2017) 78:4586–98. doi: 10.1158/0008-5472.can-17-3841 29880482

[206] Yue X Lan F Xia T . Hypoxic glioma cell-secreted exosomal miR-301a activates Wnt/β-catenin signaling and promotes radiation resistance by targeting TCEAL7. Mol Ther. (2019) 27:1939–49. doi: 10.1016/j.ymthe.2019.07.011 31402274 PMC6838947

[207] Kawano M Tanaka K Itonaga I Iwasaki T Tsumura H . Microrna-301a promotes cell proliferation via PTEN targeting in Ewing’s sarcoma cells. Int J Oncol. (2016) 48:1531–40. doi: 10.3892/ijo.2016.3379 26846737

[208] Granda-Díaz R Manterola L Hermida-Prado F Rodríguez R Santos L García-de-la-Fuente V . Targeting oncogenic functions of miR-301a in head and neck squamous cell carcinoma by PI3K/PTEN and MEK/ERK pathways. BioMed Pharmacother. (2023) 161:114512. doi: 10.1016/j.biopha.2023.114512 36931033

[209] Alonso‐Crisostomo L Trendell J Ferraresso M Bailey S Ward D Scurlock ZGL . Testicular germ cell tumor cells release microRNA‐containing extracellular vesicles that induce phenotypic and genotypic changes in cells of the tumor microenvironment. Int J Cancer. (2024) 154:372–88. doi: 10.1002/ijc.34697 37632231

[210] Gayer FA Henkel M Luft J Reichardt SD Fichtner A Legler TJ . The subtype identity of testicular cancer cells determines their immunostimulatory activity in a coculture model. Cancers. (2023) 15:2619. doi: 10.3390/cancers15092619 37174085 PMC10177190

[211] Batool A Wang Y-Q Hao X-X Chen S-R Liu Y-X . A miR-125b/CSF1-CX3CL1/tumor-associated macrophage recruitment axis controls testicular germ cell tumor growth. Cell Death Dis. (2018) 9:962. doi: 10.1038/s41419-018-1021-z 30237497 PMC6148032

[212] Shou Y Wang X Chen C Liang Y Yang C Xiao Q . Exosomal miR-301a-3p from esophageal squamous cell carcinoma cells promotes angiogenesis by inducing M2 polarization of macrophages via the PTEN/PI3K/AKT signaling pathway. Cancer Cell Int. (2022) 22:153. doi: 10.1186/s12935-022-02570-6 35436935 PMC9014619

[213] Henau OD Rausch M Winkler D Campesato LF Liu C Cymerman DH . Overcoming resistance to checkpoint blockade therapy by targeting PI3Kγ in myeloid cells. Nature. (2016) 539:443–7. doi: 10.1038/nature20554 27828943 PMC5634331

[214] Kaneda MM Messer KS Ralainirina N Li H Leem CJ Gorjestani S . PI3Kγ is a molecular switch that controls immune suppression. Nature. (2016) 539:437–42. doi: 10.1038/nature19834 27642729 PMC5479689

[215] Hong DS Postow M Chmielowski B Sullivan R Patnaik A Cohen EEW . Eganelisib, a first-in-class PI3Kγ inhibitor, in patients with advanced solid tumors: results of the phase 1/1b MARIO-1 trial. Clin Cancer Res. (2023) 29:2210–9. doi: 10.1158/1078-0432.ccr-22-3313 37000164 PMC10388696

[216] O’Connell BC Hubbard C Zizlsperger N Fitzgerald D Kutok JL Varner J . Eganelisib combined with immune checkpoint inhibitor therapy and chemotherapy in frontline metastatic triple-negative breast cancer triggers macrophage reprogramming, immune activation and extracellular matrix reorganization in the tumor microenvironment. J Immunother Cancer. (2024) 12:e009160. doi: 10.1136/jitc-2024-009160 39214650 PMC11367338

[217] Peng W Chen JQ Liu C Malu S Creasy C Tetzlaff MT . Loss of PTEN promotes resistance to T cell–mediated immunotherapy. Cancer Discov. (2016) 6:202–16. doi: 10.1158/2159-8290.cd-15-0283 26645196 PMC4744499

[218] Vidotto T Melo CM Castelli E Koti M Reis R Squire JA . Emerging role of PTEN loss in evasion of the immune response to tumors. Br J Cancer. (2020) 122:1732–43. doi: 10.1038/s41416-020-0834-6 32327707 PMC7283470

[219] Isoyama S Mori S Sugiyama D Kojima Y Tada Y Shitara K . Cancer immunotherapy with PI3K and PD-1 dual-blockade via optimal modulation of T cell activation signal. J Immunother Cancer. (2021) 9:e002279. doi: 10.1136/jitc-2020-002279 34446575 PMC8395371

[220] Ma X Yan F Deng Q Li F Lu Z Liu M . Modulation of tumorigenesis by the pro-inflammatory microRNA miR-301a in mouse models of lung cancer and colorectal cancer. Cell Discov. (2015) 1:15005. doi: 10.1038/celldisc.2015.5 27462406 PMC4860842

[221] Belge G Grobelny F Matthies C Radtke A Dieckmann K-P . Serum level of microRNA-375-3p is not a reliable biomarker of teratoma. Vivo. (2020) 34:163–8. doi: 10.21873/invivo.11757 31882475 PMC6984070

[222] Kremer L Brandenstein M Wittersheim M Koeditz B Paffenholz P Hellmich M . The combination of microRNA-371a-3p and 375-5p can distinguish viable germ cell tumor and teratoma from necrosis in postchemotherapy retroperitoneal lymph node dissection specimens. Transl Androl Urol. (2021) 10:1647655–1641655. doi: 10.21037/tau-20-1349 33968653 PMC8100847

[223] Yodkhunnatham N Pandit K Puri D Yuen KL Bagrodia A . MicroRNAs in testicular germ cell tumors: the teratoma challenge. Int J Mol Sci. (2024) 25:2156. doi: 10.3390/ijms25042156 38396829 PMC10889716

